# Comprehensive Virulence Gene Profiling of Bovine Non-*aureus* Staphylococci Based on Whole-Genome Sequencing Data

**DOI:** 10.1128/mSystems.00098-18

**Published:** 2019-03-05

**Authors:** Sohail Naushad, S. Ali Naqvi, Diego Nobrega, Christopher Luby, John P. Kastelic, Herman W. Barkema, Jeroen De Buck

**Affiliations:** a Department of Production Animal Health, Faculty of Veterinary Medicine, University of Calgary, Calgary, Alberta, Canada; b Canadian Bovine Mastitis and Milk Quality Research Network, St-Hyacinthe, Quebec, Canada; c Department of Large Animal Clinical Sciences, Western College of Veterinary Medicine, University of Saskatchewan, Saskatoon, Saskatchewan, Canada; University of Illinois at Urbana-Champaign

**Keywords:** adherence, coagulase-negative staphylococci, exoenzymes, host immune evasion, intramammary infection, iron uptake and metabolism, phenol-soluble modulins, toxins, virulence factors, whole-genome sequencing

## Abstract

Non-*aureus* staphylococci (NAS) are the most frequently isolated pathogens from intramammary infection (IMI) in dairy cattle. Virulence factors (VFs) and mechanisms by which NAS cause IMI are not fully known. Herein, we analyzed the distribution of 191 VFs in 441 genomes of 25 NAS species, after classifying VFs into functional categories: adherence (*n* = 28), exoenzymes (*n* = 21), immune evasion (*n* = 20), iron metabolism (*n* = 29), and toxins (*n* = 93). In addition to establishing VF gene profiles, associations of VF genes between and among functional categories were computed, revealing distinctive patterns of association among VFs for various NAS species. Associations were also computed for low, medium, and high somatic cell count (SCC) and clinical mastitis (CM) isolates, demonstrating distinctive patterns of associations for low SCC and CM isolates, but no differences between high SCC and CM isolates. To determine whether VF distributions had any association with SCC or CM, various clustering approaches, including complete linkages, Ward clustering, and t-distributed stochastic neighbor embedding, were applied. However, no clustering of isolates representing low SCC, medium SCC, or high SCC or CM was identified. Regression analysis to test for associations with individual VF functional categories demonstrated that each additional toxin and host immune evasion gene increased the odds of having high SCC or CM, although an overall increase in the number of VFs was not associated with increased SCC or occurrence of CM. In conclusion, we established comprehensive VF gene profiling, determined VF gene distributions and associations, calculated pathogenic potentials of all NAS species, and detected no clear link between VF genes and mastitis.

**IMPORTANCE** Non-*aureus* staphylococci (NAS) are the most frequently isolated pathogens from milk in dairy cattle worldwide. The virulence factors (VFs) and mechanisms by which these bacteria cause udder infection are not fully known. We determined the distribution and associations of 191 VFs in 25 NAS species and investigated the relationship between VFs and disease. Although the overall number of VFs was not associated with disease severity, increasing numbers of toxin and host immune evasion genes specifically were associated with more severe disease outcomes. These findings suggest that the development of disease and the interactions of VFs with the host are complex and determined by the interplay of genes rather than just the presence of virulence genes. Together, our results provide foundational genetic knowledge to other researchers to design and conduct further experiments, focusing on understanding the synergy between VFs and roles of individual NAS species in IMI and characterizing species-specific effects on udder health.

## Abstract (precis)

Non-*aureus* staphylococci (NAS) are the most frequently isolated pathogens from milk in dairy cattle worldwide. The virulence factors (VFs) and mechanisms by which these bacteria cause udder infection are not fully known. We determined the distribution and associations of 191 VFs in 25 NAS species and investigated the relationship between VFs and disease. Although the overall number of VFs was not associated with disease severity, increasing numbers of toxin and host immune evasion genes specifically were associated with more severe disease outcomes. These findings suggest that the development of disease and the interactions of VFs with the host are complex and determined by the interplay of genes rather than just the presence of virulence genes. Together, our results provide foundational genetic knowledge to other researchers to design and conduct further experiments, focusing on understanding the synergy between VFs and roles of individual NAS species in IMI and characterizing species-specific effects on udder health.

## INTRODUCTION

Non-*aureus* staphylococci (NAS), most of which are coagulase-negative staphylococci (CNS), are the most frequently isolated bacteria from bovine milk (1–3). Although NAS are often considered minor mastitis pathogens (3, 4), they are increasingly recognized as dominant pathogens of bovine mastitis worldwide (1, 3, 5). The genus *Staphylococcus* (as of October 2018) includes 53 species and 28 subspecies (http://www.bacterio.net/staphylococcus.html), of which 25 NAS species are commonly isolated from milk from dairy cows in Canada and other countries. Interspecies relationships and prevalence of these species were recently reported by our group (1, 6). However, pathogenesis of these bacteria is not fully understood. Therefore, it is not clear whether NAS should be considered commensal bacteria or opportunistic pathogens. Additionally, the effects of individual NAS species on udder health are not well characterized (7–9). Mechanisms that allow these organisms to colonize and cause mastitis are not well-known (2, 3, 10). Generally, obligate or opportunistic pathogenic bacteria sense host signals and adapt gene expression to match environmental conditions (11–13). A subset of these genes has a key role in the ability of the bacterium to cause disease (14, 15). Products of such genes that facilitate successful colonization and survival of the bacterium in a host environment and damage that host are considered pathogenicity determinants or virulence factors (VFs) (13, 15, 16). The VFs are either coded within bacterial genomes, mostly on specific genomic loci, referred to as pathogenicity islands, or coded within transmissible genetic elements that can spread among bacteria (13, 17–20). Numerous VFs are known for Staphylococcus aureus (SAU), an important pathogen of various animals, including dairy cattle, where it is recognized as a major udder pathogen, commonly associated with (sub)clinical mastitis (21, 22). Staphylococcus aureus has an array of genes involved in adhesion, invasion, and host defense evasion (23). The VFs and mechanisms by which SAU causes intramammary infections (IMI) in dairy cattle are well characterized, compared to NAS-related IMI (24–27). Few studies have focused on VFs of NAS isolated from bovine mastitis (28–31). All VFs and mechanisms by which various bovine NAS species survive, multiply, and cause disease in the host are yet to be fully identified. Thus, to determine pathogenic potential (number of VFs and their associations) of individual or closely related NAS species and to investigate whether the presence and absence of VFs have associations with occurrence of mastitis, comprehensive identification of putative VFs from the 25 most common NAS species is essential. Genome-based phylogeny of 25 bovine NAS species was recently established by our group (32), which divided NAS species into five distinct clades (A to E). Clade A contained SVI, SFL, and SSC (for NAS species and NAS species abbreviations, see “Classification and distribution of virulence factors” in Materials and Methods). Clade B included SAG, SHY, and SSC, clade C was represented by SSI. Clade D was divided into D1 (SHO, SDE, and SHA), D2 (SPA and SWA), and D3 (SEP, SCR and SCI), and clade E contained SAC, SAR, SKL, SSU, SGA, SCO, SNE, SEQ, SSA, and SXY.

In this study, genomic data for 441 isolates from 25 NAS species from 87 herds were used. Our objectives follow: (i) to identify and determine the distribution of putative VFs (pVFs) among 25 NAS species; (ii) to investigate relationships among pVFs; (iii) to identify distinct pVF associations for low, medium, and high somatic cell count (SCC) and clinical mastitis (CM) isolates; and finally (iv) to investigate the association between the presence of pVFs and CM.

## RESULTS

### Distribution and associations of VF genes involved in adherence.

**FIG 1:**
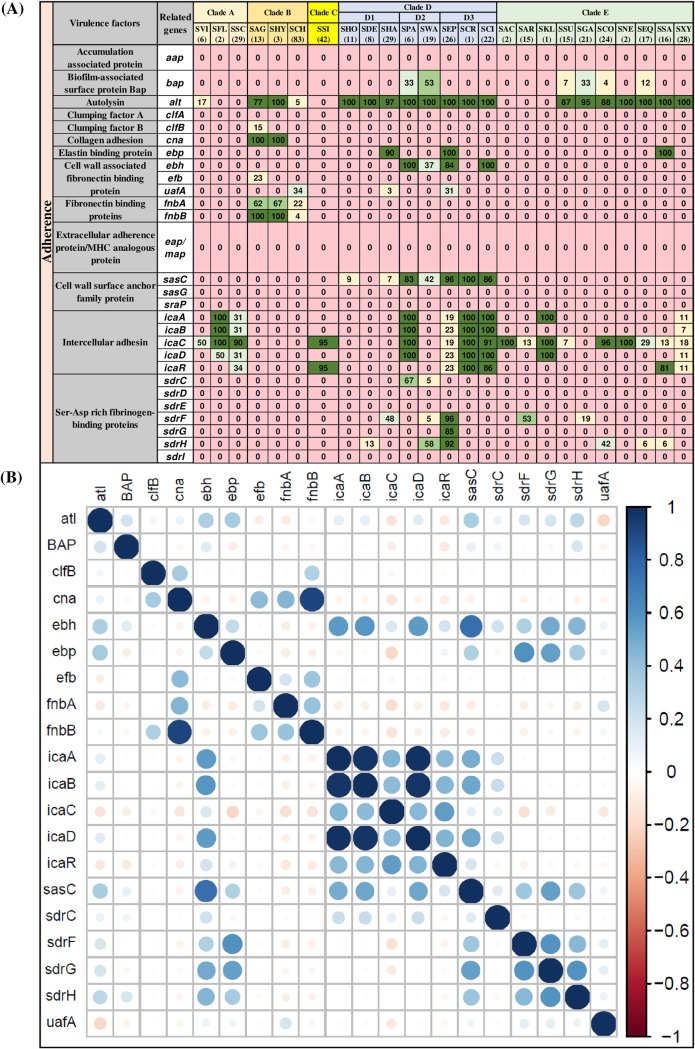
Distributions and pairwise associations of 28 adherence-related virulence factors. (A) Distribution of adherence-related virulence factors of 25 NAS species, arranged into five clades (A, B, C, D, and E) according to their placement in phylogenetic trees (32). Values and different colors within cells represent the percentage of isolates containing a given gene. The color gradient from red to dark green indicates increasing percentage of isolates containing the particular gene within species (light yellow, 1 to 25% of isolates; light blue, 26 to 50% of isolates; light green, 51 to 75% of isolates; dark green, 76 to 100% of isolates). (B) Pairwise associations of genes, computed using phi coefficients (143), with a color gradient representing the type of association (blue for positive; red for negative), while the intensity of the color and size of the circle show the strength of the association.

The 28 VFs of the adherence category tested in this study included accumulation-associated protein (*aap*), biofilm-associated surface protein Bap (*bap*), autolysin (*atl*), clumping factors (*clfA* and *clfB*), collagen adhesion (*cna*), elastin binding protein (*ebp*), fibronectin binding proteins (*ebh*, *efb*, *uafA*, *fnbA*, and *fnbB*), extracellular adherence/MHC analogous protein (*eap/map*), cell wall surface anchor family proteins (*sasC*, *sasG*, and *sasP*), intercellular adhesins (*icaA*, *icaB*, *icaC*, *icaD*, and *icaR*), and Ser-Asp-rich fibrinogen binding proteins (*sdrC*, *sdrD*, *sdrE*, *sdrF*, *sdrG*, *sdrH*, and *sdrI*) (Fig. 1A). Among these genes, *atl* was the most frequently detected gene in 20 out of 25 NAS species in at least one isolate. The *atl* gene was present in SCH, SVI, SSU, SCO, SGA, SHA, SHY, SHO, SDE, SPA, SWA, SEP, SCR, SCI, SNE, SEQ, SSA, and SXY but was not detected in isolates of SFL, SSC, SSI, SAC, SKL, and SAR. The *icaC* gene of the *ica* operon, believed to be involved in biofilm formation (33) was the second most frequent gene and was present in 17 of 25 NAS species (Fig. 1A). Other genes of the *ica* operon, namely, *icaA*, *icaB*, *icaD*, and *icaR* were detected in eight, seven, eight, and seven NAS species, respectively. Another biofilm-related gene, *bap* (encodes biofilm-associated surface protein Bap) was present in only six NAS species (Fig. 1A).

Among the clade A species, SVI contained only 2 (*alt* and *icaC*) of 28 adhesion genes, whereas SFL and SSC contained 4 and 5 genes from the *ica* operon (Fig. 1A). Among species of clade B (SAG, SHY, and SCH), *atl*, *clfB*, *cna*, *efb*, *uafA*, *fnbA*, and *fnbB* were the most widespread genes. SSI, the sole representative species of clade C (in our data), contained only *icaA* and *icaR*. Many genes from the adherence category, including *aap*, *clfA*, *eap/map*, *sasG*, *sraP*, *sdrD*, *sdrE*, and *sdrI*, were not detected in any of the 441 NAS isolates. One or more genes from the intracellular adhesin family (*ica* operon) were present in many NAS species, except species from clades B and D1. Two genes, *fnbA* and *fnbB*, responsible for encoding fibronectin binding proteins, were identified in only three species (SAG, SHY, and SCH) from clade B. The *cna* gene, which contributes to collagen adhesion, was detected in two (of three) species from clade B (SAG and SHY) only. The distributions and frequencies of other clade-specific VF genes are shown in Fig. 1A. In this study, a VF was considered present in the NAS species, even if that VF was detected only in one isolate of that particular species.

Associations between 28 adherence genes in NAS species are shown (Fig. 1B). A strong positive association was detected between *fnbB* and *cna*, with both *fnbB* and *cna* present in 100% isolates of SAG and SHY (Fig. 1A). There were also positive associations for a second fibronectin binding *fnbA* and *cna*, however, with lower intensity compared to the previous example shown for *fnbB*, corresponding to frequencies of 62 and 67% in isolates of SAG and SHY (Fig. 1A), although *cna* was present in 100% of isolates of these species. Similarly, strong positive associations were present among *icaA*, *icaB*, and *icaD* of the *ica* operon (Fig. 1B). Generally, within the adherence category, most associations were either neutral or positive; no strong negative association was detected within this category, and all negative associations were very weak.

### Distribution and associations of exoenzymes.

**FIG 2:**
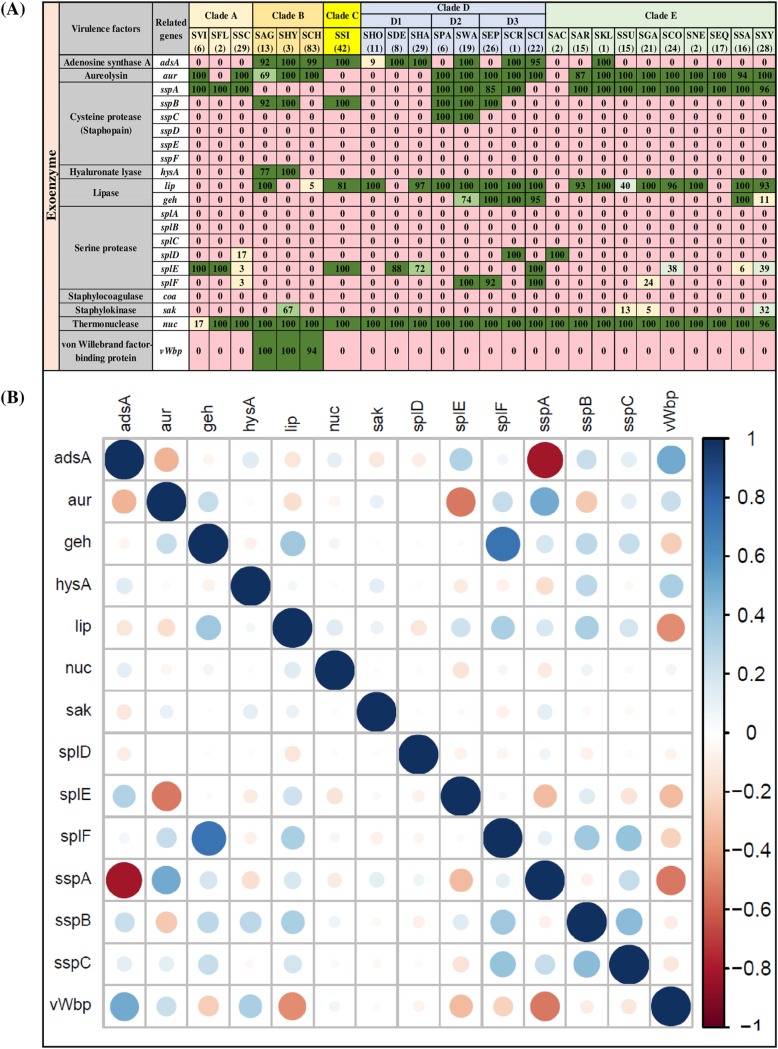
Distributions and pairwise associations of 21 exoenzyme genes in NAS species. (A) Distribution of exoenzymes genes of 25 NAS species, arranged into five clades (A, B, C, D, and E) according to their placement in phylogenetic trees (32). Values and different colors within cells represent the percentage of isolates containing a given gene. The color gradient from red to dark green indicates increasing percentage of isolates containing the particular gene within species(light yellow, 1 to 25% of isolates; light blue, 26 to 50% of isolates; light green, 51 to 75% of isolates; dark green, 76 to 100% of isolates). (B) Pairwise associations of genes, computed using phi coefficients (143), with a color gradient representing the type of association (blue for positive; red for negative), while the intensity of the color and the size of the circle show the strength of the association.

The second category of VFs (21 genes), exoenzymes (Fig. 2A), consisted of adenosine synthase A (*adsA*), aureolysin (*aur*), cysteine proteases (*sspA*, *sspB*, *sspC*, *sspD*, *sspE*, and *sspF*), hyaluronate lyase (*hysA*), lipases (*lip* and *geh*), serine proteases (*splA*, *splB*, *splC*, *splD*, *splE*, and *splF*), staphylocoagulase (*coa*), staphylokinase (*sak*), thermonuclease (*nuc*) and von Willebrand factor binding protein (*vWbp*). Thermonuclease (*nuc*) was present in almost 100% of NAS isolates, except SVI, where it was identified in 17% of isolates. The second most frequent exoenzyme genes were *aur* and *lip*, which were detected in 19 and 18 NAS species, respectively. The gene for another lipase, *geh*, was detected in six NAS species. The enzyme *adsA* was identified in all species from clades B and C and in all species from clade D, except SPA and SEP, but in only one species from clade E (SKL) (represented by a single isolate). Clade-specific distributions of enzymes were observed for *sspB*, which was identified in all isolates of SPA and SWA (species from clade D2) and *vWbp*, detected in SAG (100%), SHY (100%), and SCH (94%) species from clade B. Hyaluronate lyase (*hysA*) was present in only two clade B species (SAG and SHY). Seven of the listed exoenzymes (*sspD*, *sspE*, and *sspF* and *splA*, *splB*, *splC*, and *coa*) were not detected in any NAS species. Distributions and frequencies of each exoenzyme described above are shown (Fig. 2A). Within exoenzymes, there was a strong positive association between *splF* and *geh*, whereas there was a strong negative association between *sspA* and *adsA* (Fig. 2B).

### Distribution and associations of VF genes involved in host immune evasion.

**FIG 3:**
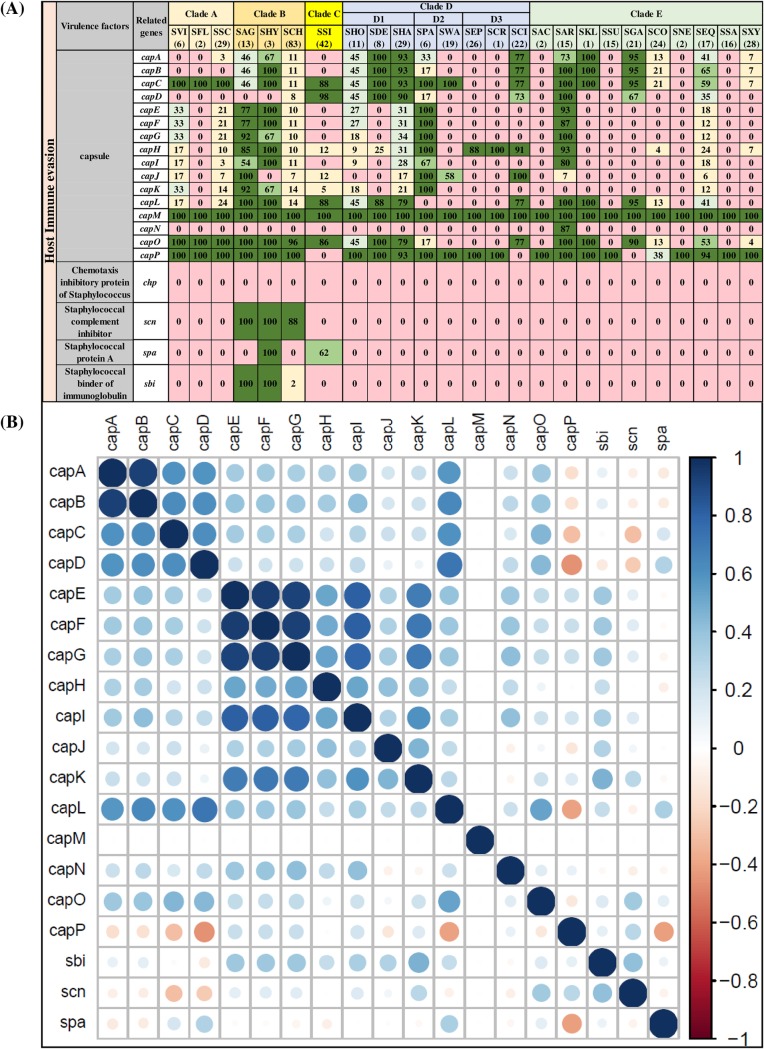
Distributions and pairwise associations of 16 host immune evasion genes. (A) Distribution of host immune evasion genes of 25 NAS species, arranged into five clades (A, B, C, D, and E) according to their placement in phylogenetic trees (32). Values and different colors within cells represent the percentage of isolates containing a given gene. The color gradient from red to dark green indicates increasing percentage of isolates containing the particular gene within species (light yellow, 1 to 25% of isolates; light blue, 26 to 50% of isolates; light green, 51 to 75% of isolates; dark green, 76 to 100% of isolates). (B) Pairwise associations of genes, computed using phi coefficients (143), with a color gradient representing the type of association (blue for positive; red for negative), while the intensity of the color and the size of the circle show the strength of the association.

The host immune evasion category consisted of 16 genes involved in capsular synthesis (*capA*, *capB*, *capC*, *capD*, *capE*, *capF*, *capG*, *capH*, *capI*, *capJ*, *capK*, *capL*, *capM*, *capN*, *capO*, and *capP*), chemotaxis inhibitory protein (*chp*), staphylococcal complement inhibitor (*scn*), staphylococcal protein A (*spa*), staphylococcal binder of immunoglobulin (*sbi*) gene. The distribution and frequencies of these VFs are shown (Fig. 3A). The *chp* gene was not detected in any NAS species. The *scn* and *sbi* genes were exclusively identified in species from clade B (SAG, SHY, and SCH) and were absent in other NAS species. The *spa* gene was present only in SHY (100%) and SSI (62%) species. Capsular genes were the most commonly detected and frequently distributed genes in this category. In this study, *cap* genes were considered present if hits were detected for either isoform *cap5* or *cap8* (34, 35). Among capsular genes, *capM* was detected in all 441 NAS isolates, and most NAS species contained ≥8 capsular genes, except SAC, SSA, and SNE which contained *capM* and *capP* only. SCH contained most of the capsular genes, except *capN*. However, except for *capM*, *capO*, and *capP*, which were detected in all isolates of SCH, frequencies of other genes were within 7 to 14% of isolates. Most capsular genes had positive associations, except *capP* which had a negative association with *capD* and *capL*. The strongest associations were between *capA* and *capB* and among *capE*, *capF*, and *capG* (Fig. 3B).

### Distribution and associations of VF genes involved in iron uptake and metabolism.

**FIG 4:**
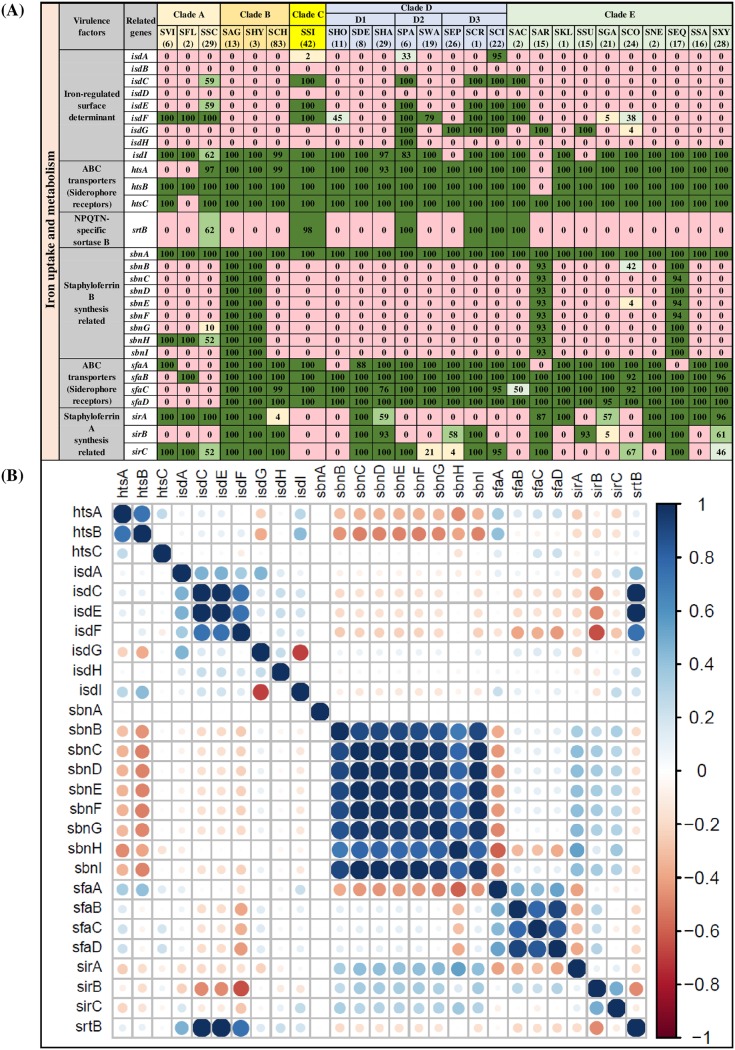
Distributions and pairwise associations of 29 virulence factors related to iron uptake and metabolism. (A) Distribution of iron uptake- and metabolism-related virulence factors of 25 NAS species, arranged into five clades (A, B, C, D, and E) according to their placement in phylogenetic trees (32). Values and different colors within cells represent the percentage of isolates containing a given gene. The color gradient from red to dark green indicates increasing percentage of isolates containing the particular gene within species (light yellow, 1 to 25% of isolates; light blue, 26 to 50% of isolates; light green, 51 to 75% of isolates; dark green, 76 to 100% of isolates). (B) Pairwise associations of genes, computed using phi coefficients (143), with a color gradient representing the type of association (blue for positive; red for negative), while the intensity of the color and the size of the circle show the strength of the association.

Twenty-nine genes involved in iron uptake and metabolism were detected, including 9 iron-regulated surface determinant genes (*isdA*, *isdB*, *isdC*, *isdD*, *isdE*, *isdF*, *isdG*, *isdH*, and *isdI*), 7 ABC transporter (also known as siderophore receptors) genes (*htsA*, *htsB*, *htsC*, *sfaA*, *sfaB*, *sfaC*, and *sfaD*), 12 staphyloferrin A and B synthesis-related genes (*sirA*, *sirB*, *sirC*, *sbnA*, *sbnB*, *sbnC*, *sbnD*, *sbnE*, *sbnF*, *sbnG*, *sbnH*, and *sbnI*) and 1 sortase B gene (*srtB*). Among 9 iron-regulated surface determinant genes, *isdI* was detected in 22 NAS species but was not present in SEP, SAR, and SSU. The *isdC*, *isdE*, and *srtB* genes were detected in SSC, SSI, SPA, SCR, SCI, and SAC only. The *isdH* gene was identified only in SPA (100%). Two genes from iron-regulated surface determinants (*isdB* and *isdD*) were not detected in any NAS species. Seven genes of ABC transporters (siderophore receptors) were uniformly distributed, with few exceptions, such as the absence of *htsA* from SVI, SFL, and SAR and the absence of *htsB* and *htsC* from SAR and SFL, respectively. Staphyloferrin A synthesis-related genes (*sirA*, *sirB*, and *sirC*) were detected more frequently than staphyloferrin B synthesis-related genes. *sbnA*, involved in staphyloferrin B synthesis, was the only gene from the iron uptake and metabolism category identified in all 441 NAS isolates. Distributions and frequencies of all 29 VFs involved in iron uptake and metabolism are shown (Fig. 4A). Both negative and positive associations were detected among VFs of iron uptake and metabolism. For instance, siderophore receptor genes (ABC transporters) were mostly positively associated (Fig. 4B). Similarly, staphyloferrin B synthesis genes also had positive associations. Negative associations were present among the ABC transporter *htsA* and *htsB* (Fig. 4B). Iron-regulated surface determinant *isdC* and *isdE* had strong positive associations, whereas *isdG* and *isdI* had a negative association (Fig. 4B). The *srtB* gene had strong associations with *isdC* and *isdE* (Fig. 4B).

### Distribution and association of toxin, type IV secretion, and phenol-soluble modulin genes.

**FIG 5:**
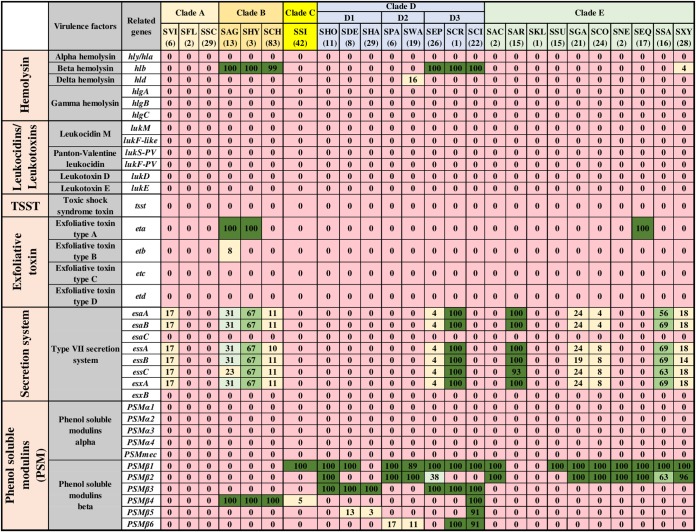
Distributions of toxin genes of different categories. Distribution of 36 toxin genes of 25 NAS species, arranged into five clades (A, B, C, D, and E) according to their placement in phylogenetic trees (32). Values and different colors within cells represent the percentage of isolates containing a given gene. The color gradient from red to dark green indicates increasing percentage of isolates containing the particular gene within species (light yellow, 1 to 25% of isolates; light blue, 26 to 50% of isolates; light green, 51 to 75% of isolates; dark green, 76 to 100% of isolates).

**FIG 6:**
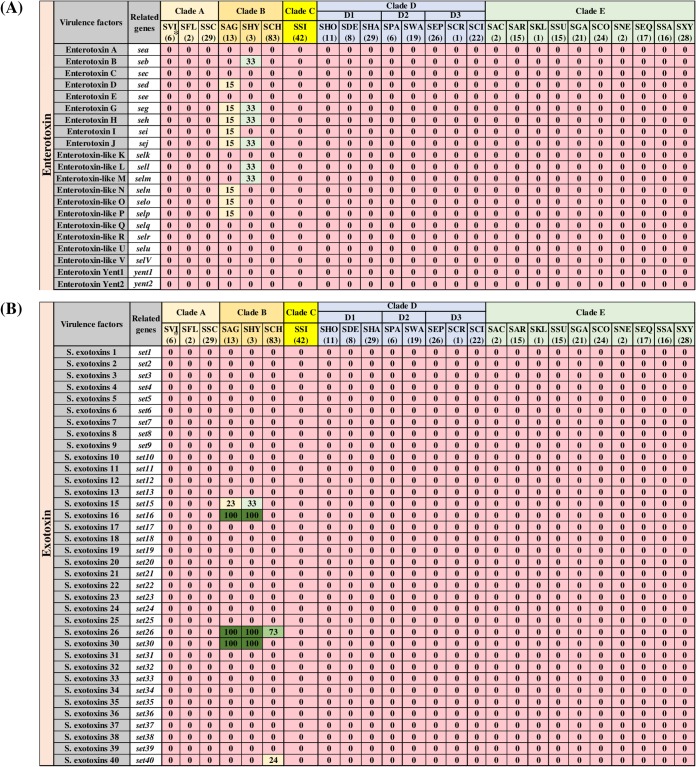
Distributions of enterotoxin and staphylococcal exotoxin genes. Distribution of 21 enterotoxin (A) and 36 staphylococcal exotoxin (B) genes of 25 NAS species, arranged into five clades (A, B, C, D, and E) according to their placement in phylogenetic trees (32). Values and different colors within cells represent the percentage of isolates containing a given gene. The color gradient from red to dark green indicates increasing percentage of isolates containing the particular gene within species (light yellow, 1 to 25% of isolates; light blue, 26 to 50% of isolates; light green, 51 to 75% of isolates; dark green, 76 to 100% of isolates).

**FIG 7:**
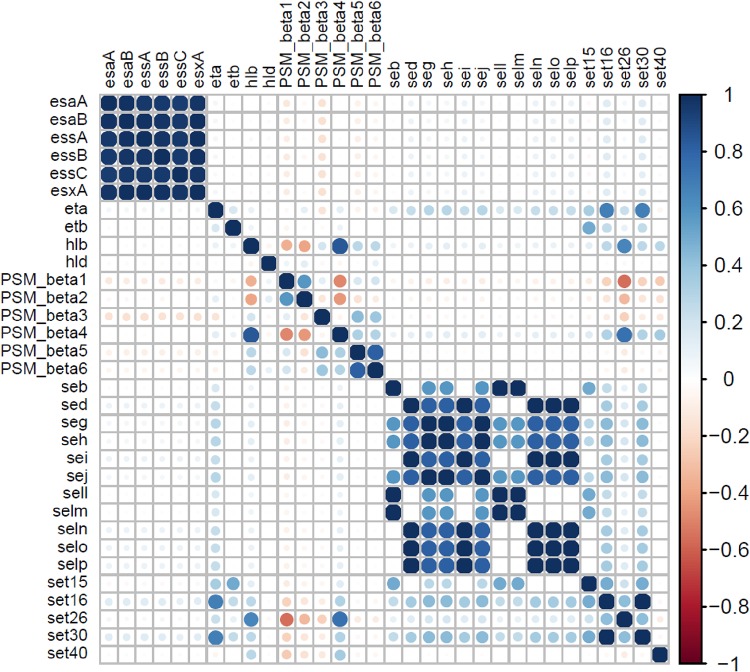
Pairwise associations of toxin genes. Pairwise associations of toxin genes, computed using the phi coefficient (143). Colors represent the type of association (blue for positive; red for negative), while the intensity of the color and the size of the circle show the strength of the correlation.

The identification and distributions of 93 toxin genes (Fig. 5 and 6) were determined in NAS isolates. Figure 5 includes 36 toxin genes from various categories, including 6 genes for alpha, beta, delta, and gamma hemolysins (*hly/hla*, *hlb*, *hld*, *hlgA*, *hlgB*, and *hlgC*), 4 genes for leukocidins, including leukocidin M (*lukM* and *lukF*-like) and Panton-Valentine leukocidins (*lukS-PV* and *lukF-PV*), 2 leukotoxins (*lukD* and *lukE*), toxic shock syndrome toxin (*tsst*), 4 exfoliative toxins (*eta*, *etb*, *etc*, and *etd*), 8 genes of type VII secretion system (*esaA*, *esaB*, *esaC*, *essA*, *essB*, *essC*, *esxA*, and *esxB*), and 11 genes for phenol-soluble modulins, including the 5 alpha modulins (*PSMα1*, *PSMα2*, *PSMα3*, *PSMα4*, and *PSMmec*) and 6 beta modulins (*PSMβ1*, *PSMβ2*, *PSMβ3*, *PSMβ4*, *PSMβ5*, and *PSMβ6*). The majority of these toxin genes were not identified in NAS species. Among hemolysin genes, beta-hemolysin (*hlb*) was identified from species of clade B (SAG, SHY, and SCH) and clade D3 (SEP, SCR, and SCI) and in 4% of isolates of SXY. The delta-hemolysin gene (*hld*) was detected in 16% of isolates of SWA only. Genes for other hemolysins (alpha and gamma), leukocidins, and leukotoxins were not detected in any NAS isolates. Similarly, *tsst*, *etc*, and *etd* were also not detected in NAS. Among the eight known genes of the type VII secretion system, *esaC* and *esxB* were not identified in NAS. The other six genes of secretion system (*esaA*, *esaB*, *essA*, *essB*, *essC*, and *esxA*) were detected in SVI, SAG, SHY, SCH, SEP, SCR, SAR, SGA, SCO, SSA, and SXY in frequencies ranging from 4% to 100%. Among PSMs, none of the PSMα genes were identified in NAS. In contrast, PSMβ genes were present in various NAS species. Detection and distribution patterns of 21 enterotoxins and 36 staphylococcal exotoxins (SETs) genes are shown (Fig. 6). These genes were not identified in the majority of the NAS isolates except for some species in clade B, especially SAG and SHY. For instance, enterotoxins, *sed*, *seg*, *seh*, *sei*, *sej*, *seln*, *selo*, and *selp* were identified in 15% of SAG isolates, whereas 33% of isolates of SHY contain *seb*, *seg*, *seh*, *sej*, *sell*, and *selm.* Similarly, from 36 SETs, only 5 SET genes were detected in 1 or more species of clade B. Of 93 toxin genes, 32 were detected in 1 or more NAS isolates (Fig. 5 and 6). There were strong positive associations among type VII secretion system genes (*esaA*, *esaB*, *essA*, *essB*, *essC*, and *esxA*) (Fig. 7). In addition, there were positive associations for some enterotoxins and staphylococcal exotoxins (Fig. 7). However, these genes were mostly identified only in SAG and SHY species (Fig. 6).

### Associations between VF genes of different categories.

**FIG 8:**
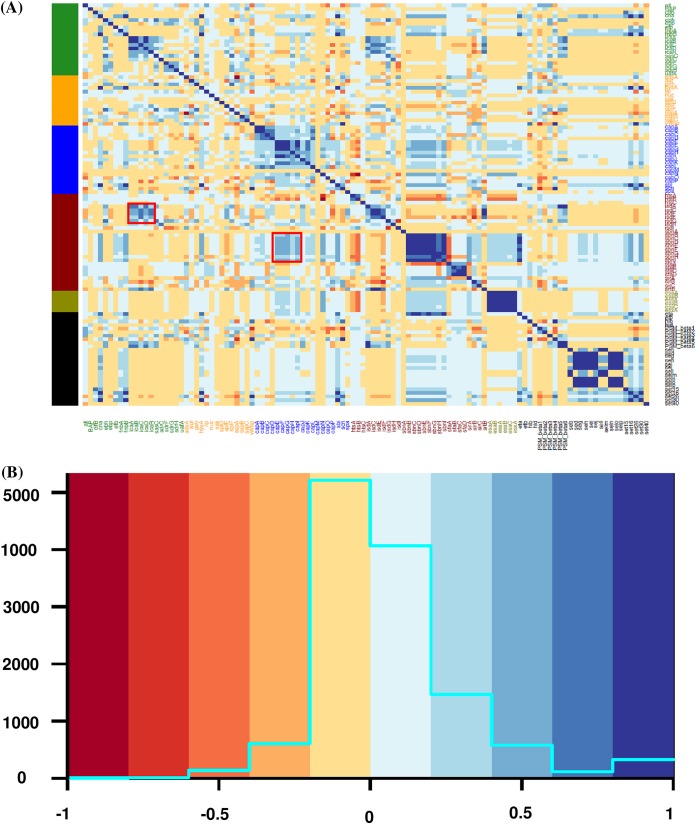
Pairwise associations and histogram of virulence factors from different functional categories. (A) Pairwise associations between virulence genes of five functional categories: adherence, exoenzymes, host immune evasion, iron uptake, and toxins. The associations were computed using the phi coefficient (143). (B) Histogram shows the total numbers of associations that fall within a particular association type. Colors in both panels represent the type of association (blue for positive; red for negative). Red boxes in panel A represent examples of clusters of positive associations between different functional categories.

**FIG 9:**
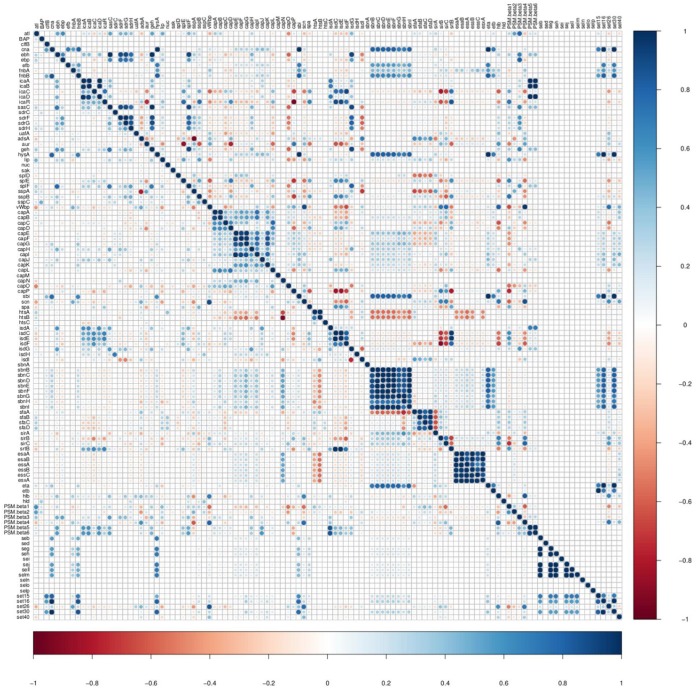
Pairwise associations and comparison of low somatic cell count isolates with clinical mastitis isolates. Mirror image showing difference in VF associations between low somatic cell count and clinical mastitis isolates. The top right triangle shows the pairwise associations of genes from clinical mastitis isolates. The bottom left triangle represents associations of virulence genes detected in low somatic cell count isolates. The associations were computed using phi coefficient (143). Colors represent the type of association (blue for positive; red for negative), while the intensity of the color and the size of the circle show the strength of the correlation.

**FIG 10:**
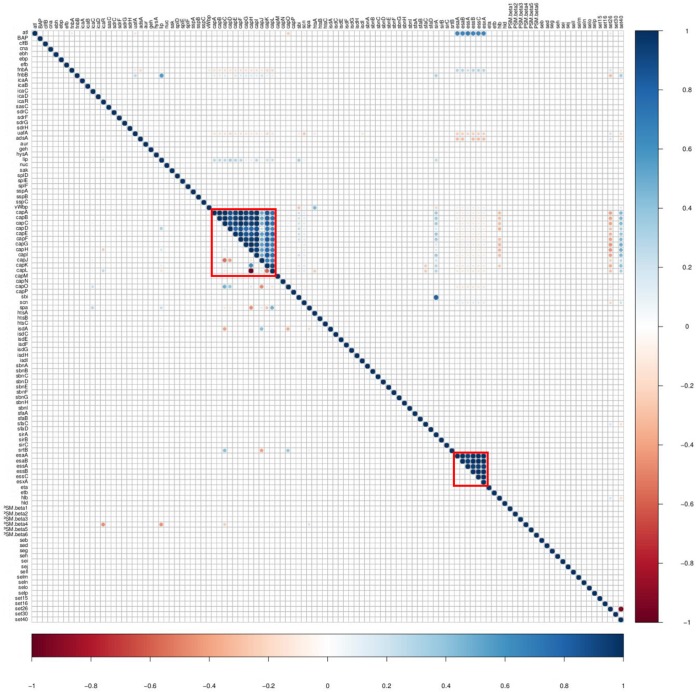
Comparison of pairwise associations between Staphylococcus chromogenes and Staphylococcus simulans. Mirror image showing differences in VF associations between two NAS species. The top right triangle shows the pairwise associations of virulence genes identified in Staphylococcus chromogenes. The bottom left triangle represents associations of virulence genes detected in Staphylococcus simulans. The distinctive associations between two species are marked with red boxes. The associations were computed using the phi coefficients (143). Colors represent the type of association (blue for positive; red for negative), while the intensity of the color and the size of the circle show the strength of the correlation.

Relationships between the VFs from five categories were investigated using an association plot in which VFs from the VF functional categories adherence, exoenzymes, host immune evasion, iron uptake and metabolism, and toxins were analyzed concurrently (Fig. 8A). Most associations among the VF functional categories were neutral or very weak (Fig. 8B), with some moderate to strong associations. For instance, adhesin genes (*icaA*, *icaB*, *icaC*, and *icaD*) were positively associated with iron regulatory genes (Fig. 8A). Similarly, capsular genes (*capE*, *capF*, *capG*, *capH*, and *capI*) were positively associated with staphyloferrin B synthesis-related genes (Fig. 8A). To assess differences in associations among four SSC classes, association plots were generated separately for isolates from low, medium, and high SCC and from CM cases. Differences in association patterns between the isolates from low SCC and isolates from CM are shown (Fig. 9). There were many distinct positive and negative association patterns in CM isolates. For example, *PSMβ5* and *PSMβ6* had very strong positive associations with the *icaA*, *icaB*, and *icaD* genes of the *ica* operon. Similarly, there were strong positive associations for staphyloferrin B synthesis-related genes and *cna*, *hysA*, and *sbi*, whereas these positive associations were absent in low SCC isolates. Also, there were many strong negative associations in CM isolates, but absent in low SCC isolates. For instance, *sirB* and *sirC* from the staphyloferrin A synthesis family had strong negative associations with collagen adhesion genes (*icaC* and *icaR*) with *isdE*, *isdF*, and *isdF*. Other important differences in gene associations between low SCC and CM isolates are shown (Fig. 9). Medium and high SCC-specific associations are shown (see Fig. S1 and S2 in the supplemental material). Finally, to discover species-specific differences in VF associations between NAS species, graphs were generated for each NAS species (*n* = 25). An example showing the species-specific difference in association between SCH and SSI is presented (Fig. 10). In this case, no strong positive and negative associations were observed in SSI, except for *capL*, which was strongly negatively correlated with *capH*, whereas in SCH, there were strong positive associations among capsular genes and type VII secretion system genes (Fig. 10). However, there were no strong negative associations in SCH VF genes.

10.1128/mSystems.00098-18.1 FIG S1 Associations of virulence genes from medium somatic cell count isolates. Mirror image showing pairwise associations of VF identified in medium somatic cell count isolates. The associations were computed using phi coefficient. Colors represent the type of association (blue for positive; red for negative), while the intensity of the color and the size of the circle show the strength of the correlation. Download FIG S1, PDF file, 0.2 MB. Copyright © 2019 Naushad et al. 2019 Naushad et al. This content is distributed under the terms of the Creative Commons Attribution 4.0 International license.

10.1128/mSystems.00098-18.2 FIG S2 Associations of virulence genes from high somatic cell count isolates. Mirror image showing pairwise associations of VF identified in high somatic cell count isolates. The associations were computed using phi coefficient. Colors represent the type of association (blue for positive; red for negative), while the intensity of the color and the size of the circle show the strength of the correlation. Download FIG S2, PDF file, 0.2 MB. Copyright © 2019 Naushad et al. 2019 Naushad et al. This content is distributed under the terms of the Creative Commons Attribution 4.0 International license.

### Association between the presence of virulence factors and mastitis.

**FIG 11:**
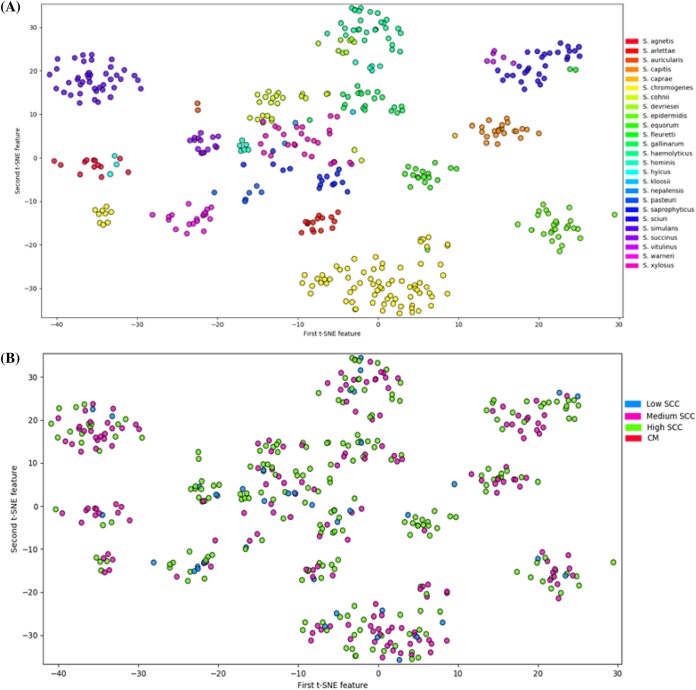
Dimensionality reduced clustering for NAS isolates determined using t-SNE. (A) Clusters labeled by 25 NAS species. (B) Clusters labeled by the inflammation severity determined in the original milk sample: low SCC, somatic cell count of ≤150,000 cells/ml; medium SCC, somatic cell count between 150,000 and 250,000 cells/ml; high SCC, somatic cell count of >250,000 cells/ml; CM, clinical mastitis.

Overall, an increase in the number of putative VFs was not associated with an increase in log SCC, although upon stratification by type of VFs, the presence of each additional toxin gene was associated with a 0.024 increase in log SCC (*P = *0.006). None of the other VF types were associated with changes in log SCC (Table 1). With inclusion of CM samples, association of numbers of VF genes were tested, with the probability of greater inflammation (i.e., higher SCC), with CM having the strongest host response. The presence of each additional VF gene associated with host immune evasion increased the odds of having a more severe immune response by 0.074 (*P = *0.003) (having one more host immune evasion gene made the isolate 1.07 times more likely to cause a more severe inflammation [i.e., increased SCC and/or CM]). Other types of VFs, however, were not associated with an increased risk of having a more severe immune response (Table 1). To investigate whether any unique pattern of the presence and absence of VF could predict disease outcome, a decision tree was generated. Although this revealed many unique patterns of VF distributions, none of these patterns were clearly associated with the level of host immune responses (low SCC, medium SCC, high SCC, and CM). In dendrograms, created by Ward clustering and complete-linkage clustering methods, placement of isolates (from low, medium, or high SCC, and CM) was polyphyletic. Isolates from all categories were randomly distributed over dendrograms (Fig. S3 and S4). No single clusters dominated by isolates from a single category were identified. Rather, most of the clustering in these dendrograms was according to species. Isolates from the same species, regardless of their isolation stage (low, medium, or high SCC or CM) were grouped together. Similarly, based on distribution patterns (generated using t-SNE algorithms), most NAS isolates clustered according to their respective species (Fig. 11A). For example, isolates from SAG, SHY, SAR, SEP, SEQ, SCI, and SSI were grouped in distinct clusters according to respective species (Fig. 11A). However, isolates from SCH were grouped in two clusters, whereas intermixing of isolates was also present for some isolates of various NAS species (Fig. 11A). Similarly, a t-SNE plot was also generated after grouping isolates into low, medium, or high SCC or CM. However, there were no unique clusters or unique patterns distinct for isolates from each of these categories (Fig. 11B). Interestingly, a comparison of clusters between (Fig. 11A and B) revealed that clustering in Fig. 11B was based on species.

**TABLE 1:** Regression model results showing the association between numbers of VFs and log SCC and inflammation severity

VF gene type	No. of VF genes	Linear regression^*a*^			Logistic regression^*b*^		
		Coefficient	Standard error	*P* value	Coefficient	Standard error	*P* value
Adherence	28	0.067	0.036	0.065	−0.020	0.039	0.607
Exoenzyme	21	0.098	0.055	0.078	0.076	0.060	0.199
Host immune evasion	20	0.045	0.023	0.054	0.074	0.025	0.003
Iron uptake	29	0.011	0.029	0.714	−0.006	0.031	0.858
Toxin	93	0.067	0.034	0.045	0.019	0.040	0.638

Total	191	0.024	0.010	0.017	0.015	0.011	0.168

a Mixed-effects linear regression using log SCC as the outcome. The coefficient represents the increase in log SCC (log number of cells/milliliter) for each additional VF detected.

b Mixed-effects ordinal logistic regression using inflammation severity as the outcome (measured as low, medium, and high SCC and clinical mastitis in order of increasing severity). The coefficient represents the increase in odds of the inflammation being more severe for each additional VF detected.

10.1128/mSystems.00098-18.3 FIG S3 Dendrogram based on virulence factors of NAS species generated from Ward clustering. The dendrogram shows the distribution of NAS isolates into different clades. The dendrogram was generated with the “AgglomerativeClustering” module, specifying four clusters (low SCC, medium SCC, high SCC, and CM) using Ward clustering (based on analysis of within-cluster variances) method. Download FIG S3, PDF file, 0.1 MB. Copyright © 2019 Naushad et al. 2019 Naushad et al. This content is distributed under the terms of the Creative Commons Attribution 4.0 International license.

10.1128/mSystems.00098-18.4 FIG S4 Dendrogram based on virulence factors of NAS species generated from complete clustering. The dendrogram shows the distribution of NAS isolates into different clades. The dendrogram was generated with the “AgglomerativeClustering” module, specifying four clusters (low SCC, medium SCC, high SCC, and CM) using complete-linkage clustering (based on maximum within-cluster distances) method. Download FIG S4, PDF file, 0.1 MB. Copyright © 2019 Naushad et al. 2019 Naushad et al. This content is distributed under the terms of the Creative Commons Attribution 4.0 International license.

## DISCUSSION

In this study, we determined the distribution of 191 *Staphylococcus* VF genes, across 441 isolates from all 25 known NAS species isolated from multiple Canadian dairy herds. Most earlier studies on VFs in NAS species were limited by the following: (i) using only a few NAS species (36), (ii) testing for a few and different VF genes (37–39), or (iii) collecting samples from very few herds (28). The first study identifying a large number of VFs in bovine-associated staphylococci was published recently (31). However, this study had only three NAS species. The 191 VFs tested in this study were grouped into five functional categories, and their distributions were determined in 441 isolates from 25 NAS species.

In this study, 28 VF genes involved in adherence and biofilm formation were tested in 25 bovine NAS species. Of these 28 adherence- and biofilm-related genes, the *ica* operon, encoding the polysaccharide intercellular adhesins (PIA), is the earliest recognized and most widely distributed genetic determinant of biofilms, mostly in human-associated NAS species (16, 40, 41). Biofilm formation starts with adhesion, initiated mostly by *atl*, followed by PIA production from the *ica* operon, which consists *of icaA*, *icaB*, *icaC*, *icaD*, and a promoter *icaR*. The *icaA* gene encodes a transferase enzyme, and *icaB* promotes deacetylation of PIA. Furthermore, *icaC* synthesizes *N*-acetylglucosamine, and *icaD* complements *icaA* action (41–43). In previous studies, there were contradictory results regarding the distribution of *ica* genes in bovine NAS species. For example, *icaA* was detected in ∼50% of NAS isolates by Tremblay et al. (37), whereas a much lower prevalence (∼5%) of *icaA* was reported by Piessens et al. (44). A difficulty in determining the role of the *ica* gene in biofilm formation by bovine NAS is that various studies have tested for different *ica* genes (37, 45, 46). We tested all genes and determined that *icaC* (17/25) was the most frequent gene, followed by *icaA*, *icaD* (8/25), and *icaB* (7/25). In contrast, most previous studies have either used *icaA* or *icaD* (or both) in biofilm studies (28, 46, 47), which may have underestimated the true prevalence of *ica*-based biofilm formation ability of NAS, especially in Canada.

None of the 25 NAS species tested in this study contained *aap* (encodes the accumulation-associated protein Aap) (48) which contributes to the primary attachment and establishment of intercellular connections in biofilms. Biofilms formed by Aap are proteinaceous in nature, in contrast to *ica*-dependent polysaccharide-based biofilms (48, 49). The absence of *aap* in our analysis, consistent with previous studies, indicated the *ica*-dependent biofilm-forming potential of our isolates (48, 49). However, distribution of *bap* (encoding biofilm-associated protein), the second most important biofilm-related gene, was sporadic. Previously, *bap* was described as a cattle-specific pathogenic factor of biofilm formation (50–52). However, in our study, this gene was detected only in species of clade D2 (SPA and SWA) and some species of clade E (SSU, SGA, SCO, and SEQ) with modest distribution frequencies (44% to 53%). Interestingly, *bap* was not detected in SCH, SSI, SHA, SXY, and SEP, the five most prevalent species causing IMI in Canada (1). However, our results are in contradiction to a recent study of three bovine NAS species, which reported the presence of *bap* in SAG and in a few isolates from SCH (31).

After adhering to the host surface, bacterial pathogens often produce a variety of exoenzymes to neutralize the immune system, damage host tissue, and degrade complex macromolecules (53) to be used as nutrients. Consistent with the results of a recent study (31), *nuc* (encodes thermonuclease) was the most frequent exoenzyme gene in our study, followed by *aur* and *sspA*. Adenosine synthase A (*adsA*) was detected mostly in species of clades B, C, and D; it converts AMP to adenosine and is considered a critical VF of SAU (54). Among proteases, cysteine proteases (*ssp* locus encoded) were more widespread than serine proteases (*spl* encoded). In SAU pathogenesis, both serine and cysteine proteases have important roles in cleaving the activity of neutrophil serine proteases, which have key roles in immune defense (55–57). Lipases were frequently distributed among NAS, except in species of clade A (Fig. 2A). The exact role of lipase in NAS pathogenicity is not understood; however, lipases are involved in release of free fatty acids (octadecanoic acid) in blood plasma (58–60). These free fatty acids affect several immune system functions and thus indirectly enhance pathogenic potential (58, 61, 62). Interestingly, SAG, SHY, and SCH contain *vWbp*, which along with *coa* and *clfA* were considered sufficient to convert commensal SSI into an invasive pathogen (63). Importantly, *coa* and *clfA* were not detected in these species in our study. However, the presence of *coa* in SAG was reported recently (31). The variable results in the coagulase test of SAG, SHY, and SCH strains (64, 65) may be due to the presence and expression of *vWbp* in these species.

Apart from exoenzyme production, encapsulation is another strategy of pathogenic bacteria to avoid host immune responses. Staphylococci, especially SAU strains, are equipped with genes enabling production of capsular polysaccharide or capsule, to protect them from phagocytosis, enhancing virulence and persistence (66–68). Among 11 known capsular polysaccharide serotypes, cap5A-P and cap8A-P are the most widespread in SAU isolated from human and bovine infections (35, 69). In our study, *capM* was the most frequent *cap* gene and was detected in all 25 NAS species, followed by *capP* and *capC*. In other studies, depending on the animal model of infection, *cap* genes either enhanced or decreased virulence of SAU. For example, capsular production enhanced virulence of SAU in murine models of bacteremia (70) and surgical wound infection (71). In contrast, there was a lower virulence of capsular gene-containing SAU in IMI (72, 73) and in catheter-induced endocarditis (74) compared to corresponding capsule gene mutants. Interestingly, based on recovery of more capsule-negative isolates from human patients with osteomyelitis, mastitis, or cystic fibrosis, the lack of a capsule was suggested to be advantageous for SAU during chronic infections (72, 75, 76). In our study, 12 capsular genes (*capA* to *capL*), in agreement with the results of a recent study (31), were present in very low frequencies (7 to 11%) among SCH isolates, the most common species of NAS in IMI worldwide. The absence of capsular genes may explain the persistence of SCH in Canadian IMI (5, 9, 77).

Apart from capsular genes, other genes common in the immune evasion cluster (IEC), such as *scn*, *chp*, *spa*, and *sbi*, have important roles in host immune evasion (78, 79). Staphylococcal complement inhibitor (*scn* product) and the chemotaxis inhibitory protein (*chp* product) are thought to be highly specific for staphylococcal isolates of human origin (80, 81). Consistent with these studies, *chp* was not detected in any of the 441 bovine NAS isolates in this study. However, *scn* was detected in species of clade B (SAG, SHY, and SCH), which is in contrast with the results of a recent study (31) who did not detect *scn* in any NAS isolates.

All 25 NAS species contained at least one gene of the iron-responsive surface determinant (*isd*) operon, with *isdI* the most frequently distributed among all NAS species, which is consistent with the results of a recent study (31). Similar to most bacteria, staphylococci must acquire iron to replicate and to sustain infections (82–84). Iron becomes scarce during infections, as body fluids are actively depleted of free iron by the host to prevent bacterial growth, a process called “nutritional immunity” (82, 83, 85). Various iron acquisition mechanisms have key roles in the pathogenesis of SAU (82). Two principal mechanisms of iron uptake and metabolism are well studied in SAU (82–84). The first mechanism involves direct uptake of iron from molecules such as heme, using *isd* genes, whereas a second mechanism involves production of siderophores called staphyloferrin A and staphyloferrin B, along with surface transporters (82, 86, 87). Both *isdI* and *isdG* are necessary genes for SAU pathogenesis (88, 89). Most ABC transporter genes (siderophore receptors) were detected in NAS species.

Staphyloferrin A synthesis genes were more widespread than staphyloferrin B and *isd* genes in NAS species; therefore, we inferred that staphyloferrin A production was the predominant mechanism of iron acquisition among NAS isolates. Staphyloferrins have also been linked to increased virulence in human NAS infections (90). However, the frequent distribution of iron uptake and metabolism-related genes among bovine NAS species (Fig. 4A) may or may not be linked to their pathogenesis, and these genes may just be required for commensalism and survival in the host.

Virulence of staphylococci, especially SAU, can be related to their ability to produce a variety of toxins. These toxins can broadly be classified as cytotoxins (hemolysins, leukotoxins, and leukocidins) and superantigenic toxins (enterotoxins, exfoliative toxins, and toxic shock syndrome toxin [TSST]). In our study of cytotoxins, beta hemolysin (*hlb*), in accordance with the results of a recent study (31), was the most frequent and mainly detected in species of clade B (SAG, SHY, and SCH) and clade D3 (SEP, SCR, and SCI), whereas delta hemolysin (*hld*) was only detected in 16% of SWA isolates. Alpha and gamma hemolysins were not detected in any of our NAS isolates. A similar study in NAS of human and bovine origin, conducted in Iran, detected the presence of *hla*, *hlb*, and *hld* and the absence of *hlg* in bovine species (91). Similarly, both *hla* and *hlb* were not detected in 76 bovine NAS isolates, although that study was limited to one herd in China (28) and genes for delta and gamma hemolysin were not included. Recently, in India, gamma hemolysin gene was present in 10 bovine NAS species; however, other hemolysin genes (*hla*, *hlb*, and *hld*) were not tested (39). None of the leukocidins (*lukM*, *lukF*-like, *lukS-PV*, and *lukF-PV*) or leukotoxins (*lukDE*) were detected in our study. However, the presence of *lukD* in one isolate of bovine SSI was reported recently (31). Recently, the *pvl* gene was reported in a few NAS isolates (4/62) in India (39). It is noteworthy that Panton-Valentine leukocidin was strongly active against human neutrophils but only weakly active against bovine neutrophils (92–94); therefore, its absence in the present study, in agreement with the results of a recent study (31), was not surprising. Inconsistencies with other studies might have been due to methodical differences and/or inclusion of isolates from different geographical niches/areas. Of four exfoliative toxin genes, *eta* was detected in all isolates of SAG, SHY, and SEQ, whereas *etb* was only in SAG (8%) and *etc* and *etd* were not detected in any NAS species in our study. Previously, *etb* was detected in SAG (31); however, *eta*, *etc*, and *etd* were not tested in this study. The *etd* was detected in SXY (95), and in SAG and SHA (39) isolated from milk from cows with mastitis. We also detected the presence of six (*esxA*, *esaA*, *esaB*, *essA*, *essB*, and *essC*) type VII secretion system (T7SS) genes in multiple species. T7SS is a protein secretion pathway in Gram-positive bacteria (96–98). In SAU, T7SS is dispensable for laboratory growth, but it is essential for virulence (97, 99, 100). The six genes detected in our study code for core components of the secretion apparatus (97, 100). On the basis of the presence of all core genes of T7SS system, we inferred that these genes may have important roles, although further characterization is needed.

Alpha-type phenol-soluble modulins (PSMs) were not detected in any NAS species, consistent with previous findings, as α-type PSMs are considered more aggressive, and have mostly been associated with more virulent SAU strains (101). According to their length, PSMs can be classified as α-type (∼20 to 25 amino acids) or β-type (43 to 45 amino acids). In our study, most NAS species contained β-type PSMs, considered less aggressive forms of PSMs. Similarly, in previous studies, SEP produced β-type but not α-type peptides (102–104). PSMs have recently emerged as a novel toxin family of staphylococci and are considered major determinants of SAU virulence (94, 102, 105). PSMs have multiple roles in staphylococcal pathogenesis, including lysis of red and white blood cells, development of biofilm, and stimulation of inflammatory responses. Since PSM genes are encoded within the core genome, they are present in virtually all *Staphylococcus* species (102, 106, 107). In contrast to PSMs being present in all staphylococci, PSMβ were not detected in three species of clade A (SVI, SFL, and SSC) and two species of clade E (SAR and SKL). However, the absence of these genes may have been due to limitations of similarity search methods. Although not true for β-type PSMs, as they have been detected by similarity searches (107), PSMα, because of their short size, often do not yield meaningful results in similarity searches (102, 107).

Much of the toxicity of staphylococci can be attributed to superantigens. Among the superantigens, toxic shock syndrome toxin (*tsst*) gene was not present in any NAS species. This was consistent with several other studies (28, 108). Although *tsst* is mainly detected in SAU, the presence of this gene in NAS has been reported (39, 109). Enterotoxins and staphylococcal exotoxins, identified originally from SAU, have been studied extensively in staphylococcal isolates originating from humans (110, 111) and animals (108, 112, 113) and in animal-derived food products (114–116). Enterotoxins produced by some staphylococcal species, apart from interrupting host immune responses, also cause food poisoning. Therefore, the presence of these toxins in milk is of great concern for the dairy industry (115, 117, 118). In most studies, prevalence of enterotoxins was determined for NAS as a group, ignoring species-specific prevalences (108, 112, 113). In our study, 22 NAS species did not contain any enteroexotoxin genes. Interestingly, all enterotoxins containing species belong to clade B of NAS (32). Unlike most other bovine NAS studies (39, 108, 113), *sea*, one of the classical enterotoxins, was not detected in any of our isolates. The absence of *sea* in NAS and in bovine NAS specifically has been reported previously (28, 119).

All isolates of each NAS species contained on average 30 or more VF genes, with the highest virulence potential (defined by total number of VFs), assigned to SAG, SHY, and SCH (clade B), largely due to exotoxins, host evasion and capsular genes, followed by SPA and SEP (clade D) and SAR and SXY (clade E). Virulence potential was lowest for SFL (clade A) and species of clade E (SAC, SSU, and SNE), which all contained <21 VF genes. However, when associations between the total number of genes and disease severity (SCC category or presence of CM) were assessed, there was no significant association of the total number of genes with disease severity (Table 1). Notwithstanding, the mere presence of genes does not guarantee their expression (120). Additionally, many other factors (e.g., host environment, nutritional status, presence of other competing microbes, and host genetics) have crucial roles in successful colonization, persistence, and pathogenicity of mammary pathogens. For example, conversion of nonpathogenic bacteria into invasive pathogens in immunocompromised hosts has been reported (13, 121). Pathogenesis is complex and often involves an organized and systematic participation of various VFs to establish disease. Often VFs complement each other to promote pathogen colonization and persistence of disease (13).

NAS isolates clustered according to their distinct species when analyzed by t-SNE and in dendrograms created by Ward clustering and complete-linkage clustering methods, indicating that isolates from each NAS species are distinct and may represent distinct pathogens. Thus, effects of NAS on udder health should not be assessed as a single group; rather, they should be characterized according to individual species. Intermixing of various NAS species observed in t-SNE plot corresponded with phylogenetic relationships of these species. For example, an intermixed cluster of SAG and SHY and of SVI, SFL, and SSC reflects their close evolutionary relationship, as evident in phylogenetic trees (32).

We also computed the difference in gene associations among NAS species and for isolates from low, medium, and high SCC and CM. Differences in associations for individual NAS species and isolates from various inflammatory responses suggest complex interplay among virulence genes in causing disease. Unraveling these interactions will be important to elucidate distinctive pathogenic mechanisms of individual NAS species and assessing species-specific effects on udder health. However, caution should be exercised in predicting synergistic functions of genes, solely based on genetic studies, as expression of one gene could have antagonistic effects on other genes (13), as demonstrated for *luk-PV* and *lukED* (122), where expression of *luk-PV* inhibited expression of *lukE* and *lukD* genes.

Although we completed the largest VF screening of all known NAS species isolated from dairy cow’s milk, our study also had some limitations. Identification of VFs based on genetic similarity could be problematic for two reasons. First, we assumed a high level of sequence conservation signified conserved function. Identification and prediction of functions of NAS VFs were extrapolated from well-characterized analogues in SAU or from NAS of human origin. However, SAU VF genes may have niche-adapted functions, impacting their roles in virulence. Second, similarity-based identification between two genes may change over time. For example, if another gene with higher similarity was established as a separate VF gene, it would alter frequency distributions in our results. Furthermore, the presence/absence of virulence-associated genes may not directly correlate with pathogenesis and severity of disease, since in addition to expression of these genes, host environment and host genetics also have major roles in disease development and progression (13). Additionally, apart from the presence or absence of VF genes, mutations in regulators, such as two-component systems, are often involved in increased virulence (13), which were not investigated in this study.

## MATERIALS AND METHODS

### Selection of isolates.

A total of 441 NAS isolates were selected for whole-genome sequencing (WGS) from a collection of 5,507 isolates as described previously (32). The milk samples were not collected specifically for this study, rather the isolates were later selected from an already existing collection. Originally, milk samples were collected in 2007 to 2008 as described previously (123), and they were stored at the Mastitis Pathogen Collection of the Canadian Bovine Mastitis and Milk Quality Research Network (CBMQRN). The SCC was measured using the Fossomatic method (Fossomatic 4000 series; Foss Electric, Hillerød, Denmark) within 5 days after sample collection (123). Milk samples were collected from 87 herds distributed over four geographic regions encompassing Atlantic Canada (Nova Scotia, Prince Edward Island, New Brunswick), Québec, Ontario, and Western Canada (Alberta) (123). Isolates included 68 NAS isolates from CM cases (defined as abnormal milk or severe clinical signs), 26 isolates with a multidrug-resistant (MDR) phenotype (124), 1 isolate per cow of uncommon species (defined as species isolated from fewer than 20 cows), and 1 randomly selected isolate per cow for all other species (Table 2).

**TABLE 2:** NAS isolates grouped according to species and inflammation groups

NAS species	No. of isolates^*a*^	% of isolates	No. of isolates in the following group:			
			Low SCC	Medium SCC	High SCC	Clinical mastitis
S. agnetis	13	2.9	2	1	8	2
S. arlettae	15 (1)	3.4	9	1	4	1
S. auricularis	2	0.5	2			
S. capitis	22	5.0	11		10	1
S. caprae	1	0.2		1		
S. chromogenes	83 (13)	18.8	33	7	20	23
S. cohnii	24 (2)	5.4	16		7	1
S. devriesei	8	1.8	4	2	1	1
S. epidermidis	27 (1)	6.1	14	2	7	4
S. equorum	17	3.9	15	1	1	
S. fleuretti	2	0.5	2			
S. gallinarum	21	4.8	12	1	7	1
S. haemolyticus	28	6.3	12	2	10	4
S. hominis	11	2.5	7	1	3	
S. hyicus	3	0.7	1		2	
S. kloosii	1	0.2	1			
S. nepalensis	2	0.5	2			
S. pasteuri	6	1.4	2	1	3	
S. saprophyticus	16	3.6	9	1	6	
S. sciuri	29	6.6	13	2	6	8
S. simulans	42 (3)	9.5	16	2	8	16
S. succinus	15	3.4	11	3	1	
S. vitulinus	6	1.4	4		2	
S. warneri	19 (1)	4.3	10	5	4	
S. xylosus	28 (5)	6.3	12	7	7	2

**Total**	**441**	**100**	**220**	**40**	**117**	**64**

a Values in parentheses indicate the numbers of MDR isolates sequenced.

### DNA extraction and whole-genome sequencing, assembly, and annotation.

Genomic DNA extraction, WGS, and genome assembly and annotation for 441 NAS isolates were performed as described previously (32, 125). Briefly, DNA was extracted with DNeasy Blood & Tissue kit (Qiagen, Toronto, ON, Canada). Quality and DNA concentrations were determined with a Qubit 2.0 fluorometer (Invitrogen, Burlington, ON, Canada). Sequencing libraries were prepared with an Illumina Nextera XT DNA library preparation kit (Illumina, San Diego, CA, USA). Paired-end sequencing (2 × 250 bp) was performed on the Illumina MiSeq platform (Illumina). An in-house pipeline, implemented in Snakemake workflow engine (126), was used to assemble and annotate genomes. Adapter sequences from the raw reads were removed using cutadapt (127), implemented in Trim Galore! 0.4.0. *De novo* assembly of these genomes was performed with Spades version 3.6.0 (128, 129). The quality of each assembled genome was assessed using Quality Assessment Tool for Genome Assemblies (QUAST) (130). Gene annotations for all genomes and gene predictions for contigs of >200 bp were done with Prokka 1.11 (131).

### Identification of virulence factors in NAS genomes.

To ensure comprehensive analysis of all VFs reported for staphylococci, a comprehensive VF data set of staphylococci (CVFS) was created (see Data Set S1 in the supplemental material). For this data set, VF sequences were obtained from publicly available databases, including the VFDB database (132), the Victors database (http://www.phidias.us/victors/), the PATRIC database (133), and phenol-soluble modulin sequences from the UniProtKB database (134). To identify VFs in NAS genomes, local “blastdbs” of individual NAS species as well as a combined blast database of all 441 NAS genomes were created with the “makeblastdb” application from BLAST+ 2.5.0 (135). All VF sequences from CVFS were blasted against NAS genomes by conducting BLASTp searches, and a single best hit for each VF query from each NAS genome was selected as a final hit. Homology between query protein sequences and blast hits was determined by calculating *H* scores (124, 136). The *H* scores between protein sequences, labeled *Ha* (where *a* represents amino acid) were calculated using the following formula: *Ha* = *VFid* × *Lm*/*Lq* (124, 136), with *VFid* representing the level of BLASTp identities between the VF query sequence and identified protein sequence, articulated as a frequency ranging from 0 to 1, *Lm* representing the length of the matching sequence from the hit, and *Lq* denoting the length of the query sequence.

10.1128/mSystems.00098-18.5 DATA SET S1 Sequences of virulence factors. Download Data Set S1, TXT file, 0.1 MB. Copyright © 2019 Naushad et al. 2019 Naushad et al. This content is distributed under the terms of the Creative Commons Attribution 4.0 International license.

A cutoff 30% sequence similarity and 50% query length coverage were used for initial searches (124, 137–139). All genomic hits that met the minimum cutoff for each individual query were selected at this stage. A final blast hit table containing all possible hits for all query sequences from all NAS genomes was imported into R v.3.4.2 (140). Hits from each query sequence were then arranged according to *Ha* score, using “dplyr” version 0.7.2 in R (141). From this list, only hits with the highest *Ha* score (highest sequence similarity and query length coverage) were selected as potential VFs in NAS genomes and the rest were discarded. In order to prevent one VF query returning hits to two different genes within a particular genome, only the highest scoring hit selected based on *Ha* score was retained (124). After identification of putative NAS VFs, to confirm orthology between identified putative NAS VF and CVFS sequences, reciprocal blast searches between putative NAS VFs and the CVFS database were performed (142). Putative NAS VF sequences that failed to match with corresponding VFs from the CVFS database as the best hit in reciprocal blast searches were not considered true orthologues and were excluded from further analyses.

### Classification and distribution of virulence factors.

A total of 191 VFs were classified into five functional categories: adherence (*n = *28; Fig. 1A), exoenzymes (*n = *21; Fig. 2A), host immune evasion (*n = *20; Fig. 3A), iron uptake and metabolism (*n = *29; Fig. 4A), and toxins (hemolysin, leukocidins, leukotoxins, toxic shock syndrome toxin, exfoliative toxins, type VII secretion system genes, phenol-soluble modulins, enterotoxins, and exotoxins) (*n = *93; Fig. 5 and 6). Distributions of each of these VFs in all NAS isolates were determined by calculating the proportion of isolates in which they were identified.

For ease of reporting, 25 non-*aureus* staphylococcal species were abbreviated as follows: Staphylococcus agnetis *=* SAG, Staphylococcus arlettae = SAR, Staphylococcus auricularis *=* SAC, Staphylococcus capitis = SCI, Staphylococcus caprae = SCR, Staphylococcus chromogenes = SCH, Staphylococcus cohnii = SCO, Staphylococcus devriesei = SDE, Staphylococcus epidermidis = SEP, Staphylococcus equorum = SEQ, Staphylococcus fleurettii = SFL, Staphylococcus gallinarum = SGA, Staphylococcus haemolyticus = SHA, Staphylococcus hominis = SHO, Staphylococcus hyicus = SHY, Staphylococcus kloosii = SKL, Staphylococcus nepalensis = SNE, Staphylococcus pasteuri = SPA, Staphylococcus saprophyticus = SSA, Staphylococcus sciuri = SSC, Staphylococcus simulans = SSI, Staphylococcus succinus = SSU, Staphylococcus vitulinus = SVI, Staphylococcus warneri = SWA, and Staphylococcus xylosus = SXY.

In the figures (Fig. 1A, 2A, 3A, 4A, 5, and 6), these 25 NAS species are grouped into five distinct clades, according to phylogenetic placement (32).

### Associations between virulence factors.

Plots were generated to determine associations among genes of five VF functional categories and between VF functional categories. Matrices of associations among VFs were computed using the phi coefficient (*vcd* package) in R v.3.4.2 (143). Before generating plots, VFs not identified in any NAS isolates were excluded from the data set. For visualization, resulting matrices were plotted using the “corrplot” package in R (144). Association plots were also generated, after classifying isolates into four SCC classes defined according to the inflammation severity score from 1 to 4, where 1 corresponds to low SCC (< 150,000 cells/ml), 2 is average SCC (150,000 – 250,000 cells/ml), 3 is high SCC (>250,000 cells/ml), and 4 corresponds to isolates from clinical cases of mastitis. In all association plots, positive associations are shown in blue, and negative associations are shown in red. Positive and negative associations were defined as the chance that if one gene was identified, then the other gene would be more or less likely identified in the same isolates, respectively.

### Association between the presence of virulence factors and mastitis.

Relationships between four SCC classes and VFs were examined after dichotomizing genes by presence or absence in all isolates. Mixed-effects ordinal logistic regression models were used to compare the odds of having an increased inflammation severity score with an increase in the total number of VFs. Ordinal logistic regression models the log odds of having more severe inflammation compared to a reference category. The models had the form:

$$\text{ln }({\text{odds}}_{s})=\kappa_{s}-\beta X-\mu_{i}$$

where odds*_s_* is the odds of interest and represents the probability of score ≤ *s* divided by the probability of score > *s*, κ*_s_* is the odds of having score = *s* divided by the odds of having score ≠ *s*, β is the regression coefficient associated with either the total number of virulence factors or the number of virulence factors within a given functional category, and μ*_i_* is the random-effects term to account for within-herd correlations.

A mixed-effects linear regression model was used to determine the association between the total number of virulence factors or the number of virulence factors within a given functional category and the natural logarithm of the SCC of the sample. This model had the form:

$$\text{ln }\left(\text{SCC}\right)=\beta X+\varepsilon +\mu_{i}$$

where β is the regression coefficient associated with either the total number of virulence factors or the number of virulence factors within a given functional category, μ*_i_* is the random-effects term to account for within-herd correlations, and ε is the residual error.

Significance was assessed using a cutoff *P* of *<*0.05, and the strength of the association and model fit were assessed using the log likelihood for the ordinal logistic regression models, and the adjusted multiple-R^2^ for linear regression models. All statistical analyses were conducted using the “ordinal” package (145) in R v.3.4.2 (140). To determine whether VF distributions were associated with four SCC classes, agglomerative dendrograms were generated with the “AgglomerativeClustering” module, specifying four clusters (low SCC, medium SCC, high SCC, and CM) using either Ward clustering (based on analysis of within-cluster variances) or complete-linkage clustering (based on maximum within-cluster distances) methods. After dendrograms were generated, labels of SCC level were applied, and their distribution among four clusters was visually examined. Clustering was assessed using the “Scikit-Learn” module in Python 3.6 (146). A decision tree based on the presence or absence of VFs was also constructed using the “DecisionTreeClassifier” module in “Scikit-Learn” (146). A tree of depth 9 was used to determine whether specific combinations of VFs could be used to predict CM. Model generalizability was assessed by calculating the classification accuracy of the model for the training set and comparing it to the accuracy of the model for a previously unseen validation set. To visualize similarities in VF distributions between isolates, t-distributed stochastic neighbor embedding (t-SNE) (147) was conducted using the “manifold.t-SNE” module within “Scikit-Learn” (146). Distributions were reduced to two dimensions and plotted such that distances between points were proportional to relative differences between isolates. Plots were labeled with CM or NAS species and visually examined to find patterns in clusters in relation to either label.

### Conclusions.

On the basis of whole-genome sequencing (WGS) data of 441 distinct NAS isolates, we established a comprehensive VF gene (*n = *191) profile of 25 NAS species and determined associations among VF genes. The VF profiles of various NAS species and their VF gene associations differed, indicating their existence as distinct pathogens. Isolates from the same species usually had the same virulence potential, with variation within species being smaller than variation between species. The variation of VF genes among isolates of the same species may represent evolution and adaptation to distinct niches or environments within the host. We also investigated the link between VF genes and inflammatory response of the mammary gland but failed to detect a clear link between VF genes and mastitis. Regardless, comprehensive studies such as ours provide foundational genetic knowledge to design and conduct further experiments, focusing on understanding the synergy between VF and roles of individual NAS species in IMI and characterizing species-specific effects on udder health. To the best of our knowledge, this was the first study to determine the distributions of 191 virulence genes in 25 NAS species isolated from bovine IMI.

### Data availability.

All whole-genome sequencing data used in this study are available (with no restrictions) from NCBI under BioProject ID PRJNA342349.

## References

[1] Condas LAZ, De Buck J, Nobrega DB, Carson DA, Naushad S, De Vliegher S, Zadoks RN, Middleton JR, Dufour S, Kastelic JP, Barkema HW 2017 Prevalence of non-*aureus* staphylococci species causing intramammary infections in Canadian dairy herds. J Dairy Sci 100:5592–5612. doi:10.3168/jds.2016-12478. 28527793

[2] Vanderhaeghen W, Piepers S, Leroy F, Van Coillie E, Haesebrouck F, De Vliegher S 2015 Identification, typing, ecology and epidemiology of coagulase negative staphylococci associated with ruminants. Vet J 203:44–51. doi:10.1016/j.tvjl.2014.11.001. 25467994

[3] Pyörälä S, Taponen S 2009 Coagulase-negative staphylococci—emerging mastitis pathogens. Vet Microbiol 134:3–8. doi:10.1016/j.vetmic.2008.09.015. 18848410

[4] Vanderhaeghen W, Piepers S, Leroy F, Van Coillie E, Haesebrouck F, De Vliegher S 2014 Effect, persistence, and virulence of coagulase-negative *Staphylococcus* species associated with ruminant udder health. J Dairy Sci 97:5275–5293. doi:10.3168/jds.2013-7775. 24952781

[5] De Visscher A, Piepers S, Haesebrouck F, De Vliegher S 2016 Intramammary infection with coagulase-negative staphylococci at parturition: species-specific prevalence, risk factors, and effect on udder health. J Dairy Sci 99:6457–6469. doi:10.3168/jds.2015-10458. 27236763

[6] Condas LAZ, De Buck J, Nobrega DB, Carson DA, Roy JP, Keefe GP, DeVries TJ, Middleton JR, Dufour S, Barkema HW 2017 Distribution of non-aureus staphylococci species in udder quarters with low and high somatic cell count, and clinical mastitis. J Dairy Sci 100:5613–5627. doi:10.3168/jds.2016-12479. 28456402

[7] Isaac P, Bohl LP, Breser ML, Orellano MS, Conesa A, Ferrero MA, Porporatto C 2017 Commensal coagulase-negative *Staphylococcus* from the udder of healthy cows inhibits biofilm formation of mastitis-related pathogens. Vet Microbiol 207:259–266. doi:10.1016/j.vetmic.2017.05.025. 28757033

[8] Petzer IM, Karzis J, Donkin EF, Webb EC, Etter EMC 2017 Validity of somatic cell count as indicator of pathogen-specific intramammary infections. J S Afr Vet Assoc 88:e1–e10. doi:10.4102/jsava.v88i0.1465. PMC6138137 28470079

[9] De Visscher A, Piepers S, Haesebrouck F, Supré K, De Vliegher S 2017 Coagulase-negative *Staphylococcus* species in bulk milk: prevalence, distribution, and associated subgroup- and species-specific risk factors. J Dairy Sci 100:629–642. doi:10.3168/jds.2016-11476. 27865514

[10] Taponen S, Liski E, Heikkilä AM, Pyörälä S 2017 Factors associated with intramammary infection in dairy cows caused by coagulase-negative staphylococci, *Staphylococcus aureus*, *Streptococcus uberis*, *Streptococcus dysgalactiae*, *Corynebacterium bovis*, or *Escherichia coli*. J Dairy Sci 100:493–503. doi:10.3168/jds.2016-11465. 28341052

[11] Bakour S, Sankar SA, Rathored J, Biagini P, Raoult D, Fournier PE 2016 Identification of virulence factors and antibiotic resistance markers using bacterial genomics. Future Microbiol 11:455–466. doi:10.2217/fmb.15.149. 26974504

[12] Didelot X, Walker AS, Peto TE, Crook DW, Wilson DJ 2016 Within-host evolution of bacterial pathogens. Nat Rev Microbiol 14:150–162. doi:10.1038/nrmicro.2015.13. 26806595 PMC5053366

[13] Diard M, Hardt WD 2017 Evolution of bacterial virulence. FEMS Microbiol Rev 41:679–697. doi:10.1093/femsre/fux023. 28531298

[14] Malachowa N, Whitney AR, Kobayashi SD, Sturdevant DE, Kennedy AD, Braughton KR, Shabb DW, Diep BA, Chambers HF, Otto M, DeLeo FR 2011 Global changes in *Staphylococcus aureus* gene expression in human blood. PLoS One 6:e18617. doi:10.1371/journal.pone.0018617. 21525981 PMC3078114

[15] Thomas MS, Wigneshweraraj S 2014 Regulation of virulence gene expression. Virulence 5:832–834. doi:10.1080/21505594.2014.995573. 25603428 PMC4601333

[16] Soumya KR, Philip S, Sugathan S, Mathew J, Radhakrishnan EK 2017 Virulence factors associated with coagulase negative staphylococci isolated from human infections. 3 Biotech 7:140. doi:10.1007/s13205-017-0753-2. PMC5462657 28593524

[17] Novick RP, Ram G 2017 Staphylococcal pathogenicity islands — movers and shakers in the genomic firmament. Curr Opin Microbiol 38:197–204. doi:10.1016/j.mib.2017.08.001. 29100762 PMC5884141

[18] Moon BY, Park JY, Hwang SY, Robinson DA, Thomas JC, Fitzgerald JR, Park YH, Seo KS 2015 Phage-mediated horizontal transfer of a Staphylococcus aureus virulence-associated genomic island. Sci Rep 5:9784. doi:10.1038/srep09784. 25891795 PMC4402969

[19] Novick RP, Ram G 2016 The floating (pathogenicity) island: a genomic dessert. Trends Genet 32:114–126. doi:10.1016/j.tig.2015.11.005. 26744223 PMC4733582

[20] Novick RP, Christie GE, Penades JR 2010 The phage-related chromosomal islands of Gram-positive bacteria. Nat Rev Microbiol 8:541–551. doi:10.1038/nrmicro2393. 20634809 PMC3522866

[21] Gao J, Barkema HW, Zhang L, Liu G, Deng Z, Cai L, Shan R, Zhang S, Zou J, Kastelic JP, Han B 2017 Incidence of clinical mastitis and distribution of pathogens on large Chinese dairy farms. J Dairy Sci 100:4797–4806. doi:10.3168/jds.2016-12334. 28434736

[22] Levison LJ, Miller-Cushon EK, Tucker AL, Bergeron R, Leslie KE, Barkema HW, DeVries TJ 2016 Incidence rate of pathogen-specific clinical mastitis on conventional and organic Canadian dairy farms. J Dairy Sci 99:1341–1350. doi:10.3168/jds.2015-9809. 26686728

[23] Foster TJ, Geoghegan JA, Ganesh VK, Höök M 2014 Adhesion, invasion and evasion: the many functions of the surface proteins of *Staphylococcus aureus*. Nat Rev Microbiol 12:49–62. doi:10.1038/nrmicro3161. 24336184 PMC5708296

[24] Kummel J, Stessl B, Gonano M, Walcher G, Bereuter O, Fricker M, Grunert T, Wagner M, Ehling-Schulz M 2016 *Staphylococcus aureus* entrance into the dairy chain: tracking *S. aureus* from dairy cow to cheese. Front Microbiol 7:1603. doi:10.3389/fmicb.2016.01603. 27790200 PMC5061776

[25] Kosciuczuk EM, Lisowski P, Jarczak J, Majewska A, Rzewuska M, Zwierzchowski L, Bagnicka E 2017 Transcriptome profiling of staphylococci-infected cow mammary gland parenchyma. BMC Vet Res 13:161. doi:10.1186/s12917-017-1088-2. 28587645 PMC5477815

[26] Capra E, Cremonesi P, Pietrelli A, Puccio S, Luini M, Stella A, Castiglioni B 2017 Genomic and transcriptomic comparison between *Staphylococcus aureus* strains associated with high and low within herd prevalence of intra-mammary infection. BMC Microbiol 17:21. doi:10.1186/s12866-017-0931-8. 28103794 PMC5247818

[27] Cremonesi P, Pozzi F, Raschetti M, Bignoli G, Capra E, Graber HU, Vezzoli F, Piccinini R, Bertasi B, Biffani S, Castiglioni B, Luini M 2015 Genomic characteristics of *Staphylococcus aureus* strains associated with high within-herd prevalence of intramammary infections in dairy cows. J Dairy Sci 98:6828–6838. doi:10.3168/jds.2014-9074. 26233457

[28] Xu J, Tan X, Zhang X, Xia X, Sun H 2015 The diversities of staphylococcal species, virulence and antibiotic resistance genes in the subclinical mastitis milk from a single Chinese cow herd. Microb Pathog 88:29–38. doi:10.1016/j.micpath.2015.08.004. 26276706

[29] Felipe V, Morgante CA, Somale PS, Varroni F, Zingaretti ML, Bachetti RA, Correa SG, Porporatto C 2017 Evaluation of the biofilm forming ability and its associated genes in *Staphylococcus* species isolates from bovine mastitis in Argentinean dairy farms. Microb Pathog 104:278–286. doi:10.1016/j.micpath.2017.01.047. 28131956

[30] Gomes F, Saavedra MJ, Henriques M 2016 Bovine mastitis disease/pathogenicity: evidence of the potential role of microbial biofilms. Pathog Dis 74:ftw006. doi:10.1093/femspd/ftw006. 26772653

[31] Åvall-Jääskeläinen S, Taponen S, Kant R, Paulin L, Blom J, Palva A, Koort J 2018 Comparative genome analysis of 24 bovine-associated *Staphylococcus* isolates with special focus on the putative virulence genes. PeerJ 6:e4560. doi:10.7717/peerj.4560. 29610707 PMC5880176

[32] Naushad S, Barkema HW, Luby C, Condas LA, Nobrega DB, Carson DA, De Buck J 2016 Comprehensive phylogenetic analysis of bovine non-aureus staphylococci species based on whole-genome sequencing. Front Microbiol 7:1990. doi:10.3389/fmicb.2016.01990. 28066335 PMC5168469

[33] Nourbakhsh F, Namvar AE 2016 Detection of genes involved in biofilm formation in *Staphylococcus aureus* isolates. GMS Hyg Infect Control 11:Doc07. doi:10.3205/dgkh000267. 27303652 PMC4804124

[34] O'Riordan K, Lee JC 2004 *Staphylococcus aureus* capsular polysaccharides. Clin Microbiol Rev 17:218–234. doi:10.1128/CMR.17.1.218-234.2004. 14726462 PMC321462

[35] Salimena AP, Lange CC, Camussone C, Signorini M, Calvinho LF, Brito MA, Borges CA, Guimaraes AS, Ribeiro JB, Mendonca LC, Piccoli RH 2016 Genotypic and phenotypic detection of capsular polysaccharide and biofilm formation in *Staphylococcus aureus* isolated from bovine milk collected from Brazilian dairy farms. Vet Res Commun 40:97–106. doi:10.1007/s11259-016-9658-5. 27255108

[36] Bochniarz M, Adaszek Ł, Dzięgiel B, Nowaczek A, Wawron W, Dąbrowski R, Szczubiał M, Winiarczyk S 2016 Factors responsible for subclinical mastitis in cows caused by *Staphylococcus chromogenes* and its susceptibility to antibiotics based on bap, fnbA, eno, mecA, tetK, and ermA genes. J Dairy Sci 99:9514–9520. doi:10.3168/jds.2016-11723. 27692714

[37] Tremblay YD, Lamarche D, Chever P, Haine D, Messier S, Jacques M 2013 Characterization of the ability of coagulase-negative staphylococci isolated from the milk of Canadian farms to form biofilms. J Dairy Sci 96:234–246. doi:10.3168/jds.2012-5795. 23141829

[38] Simojoki H, Hyvönen P, Plumed Ferrer C, Taponen S, Pyörälä S 2012 Is the biofilm formation and slime producing ability of coagulase-negative staphylococci associated with the persistence and severity of intramammary infection? Vet Microbiol 158:344–352. doi:10.1016/j.vetmic.2012.02.031. 22424866

[39] Mahato S, Mistry HU, Chakraborty S, Sharma P, Saravanan R, Bhandari V 2017 Identification of variable traits among the methicillin resistant and sensitive coagulase negative staphylococci in milk samples from mastitic cows in India. Front Microbiol 8:1446. doi:10.3389/fmicb.2017.01446. 28824577 PMC5534481

[40] O'Gara JP 2007 ica and beyond: biofilm mechanisms and regulation in *Staphylococcus epidermidis* and *Staphylococcus aureus*. FEMS Microbiol Lett 270:179–188. doi:10.1111/j.1574-6968.2007.00688.x. 17419768

[41] McKenney D, Hubner J, Muller E, Wang Y, Goldmann DA, Pier GB 1998 The ica locus of *Staphylococcus epidermidis* encodes production of the capsular polysaccharide/adhesin. Infect Immun 66:4711–4720. 9746568 10.1128/iai.66.10.4711-4720.1998 PMC108579

[42] Foka A, Katsikogianni MG, Anastassiou ED, Spiliopoulou I, Missirlis YF 2012 The combined effect of surface chemistry and flow conditions on *Staphylococcus epidermidis* adhesion and ica operon expression. Eur Cell Mater 24:386–402. doi:10.22203/eCM.v024a28. 23160991

[43] Ninin E, Caroff N, Espaze E, Maraillac J, Lepelletier D, Milpied N, Richet H 2006 Assessment of ica operon carriage and biofilm production in *Staphylococcus epidermidis* isolates causing bacteraemia in bone marrow transplant recipients. Clin Microbiol Infect 12:446–452. doi:10.1111/j.1469-0691.2006.01382.x. 16643521

[44] Piessens V, De Vliegher S, Verbist B, Braem G, Van Nuffel A, De Vuyst L, Heyndrickx M, Van Coillie E 2012 Characterization of coagulase-negative staphylococcus species from cows' milk and environment based on bap, icaA, and mecA genes and phenotypic susceptibility to antimicrobials and teat dips. J Dairy Sci 95:7027–7038. doi:10.3168/jds.2012-5400. 22999285

[45] Schonborn S, Wente N, Paduch JH, Kromker V 2017 In vitro ability of mastitis causing pathogens to form biofilms. J Dairy Res 84:198–201. doi:10.1017/S0022029917000218. 28524019

[46] Srednik ME, Tremblay YDN, Labrie J, Archambault M, Jacques M, Fernandez Cirelli A, Gentilini ER 2017 Biofilm formation and antimicrobial resistance genes of coagulase-negative staphylococci isolated from cows with mastitis in Argentina. FEMS Microbiol Lett 364:fnx001. doi:10.1093/femsle/fnx001. 28087612

[47] Rumi MV, Huguet MJ, Bentancor AB, Gentilini ER 2013 The icaA gene in staphylococci from bovine mastitis. J Infect Dev Ctries 7:556–560. doi:10.3855/jidc.2670. 23857391

[48] Schaeffer CR, Woods KM, Longo GM, Kiedrowski MR, Paharik AE, Buttner H, Christner M, Boissy RJ, Horswill AR, Rohde H, Fey PD 2015 Accumulation-associated protein enhances *Staphylococcus epidermidis* biofilm formation under dynamic conditions and is required for infection in a rat catheter model. Infect Immun 83:214–226. doi:10.1128/IAI.02177-14. 25332125 PMC4288872

[49] Büttner H, Mack D, Rohde H 2015 Structural basis of *Staphylococcus epidermidis* biofilm formation: mechanisms and molecular interactions. Front Cell Infect Microbiol 5:14. doi:10.3389/fcimb.2015.00014. 25741476 PMC4330918

[50] Cucarella C, Solano C, Valle J, Amorena B, Lasa I, Penades JR 2001 Bap, a *Staphylococcus aureus* surface protein involved in biofilm formation. J Bacteriol 183:2888–2896. doi:10.1128/JB.183.9.2888-2896.2001. 11292810 PMC99507

[51] Vautor E, Abadie G, Pont A, Thiery R 2008 Evaluation of the presence of the bap gene in *Staphylococcus aureus* isolates recovered from human and animals species. Vet Microbiol 127:407–411. doi:10.1016/j.vetmic.2007.08.018. 17881161

[52] Cucarella C, Tormo MA, Ubeda C, Trotonda MP, Monzon M, Peris C, Amorena B, Lasa I, Penades JR 2004 Role of biofilm-associated protein Bap in the pathogenesis of bovine *Staphylococcus aureus*. Infect Immun 72:2177–2185. doi:10.1128/IAI.72.4.2177-2185.2004. 15039341 PMC375157

[53] Arnosti C 2011 Microbial extracellular enzymes and the marine carbon cycle. Annu Rev Mar Sci 3:401–425. doi:10.1146/annurev-marine-120709-142731. 21329211

[54] Thammavongsa V, Kern JW, Missiakas DM, Schneewind O 2009 *Staphylococcus aureus* synthesizes adenosine to escape host immune responses. J Exp Med 206:2417–2427. doi:10.1084/jem.20090097. 19808256 PMC2768845

[55] Paharik AE, Salgado-Pabon W, Meyerholz DK, White MJ, Schlievert PM, Horswill AR 2016 The Spl serine proteases modulate *Staphylococcus aureus* protein production and virulence in a rabbit model of pneumonia. mSphere 1:e00208-16. doi:10.1128/mSphere.00208-16. PMC5061998 27747296

[56] Stapels DA, Kuipers A, von Kockritz-Blickwede M, Ruyken M, Tromp AT, Horsburgh MJ, de Haas CJ, van Strijp JA, van Kessel KP, Rooijakkers SH 2016 *Staphylococcus aureus* protects its immune-evasion proteins against degradation by neutrophil serine proteases. Cell Microbiol 18:536–545. doi:10.1111/cmi.12528. 26418545

[57] Rudack C, Sachse F, Albert N, Becker K, von Eiff C 2009 Immunomodulation of nasal epithelial cells by *Staphylococcus aureus*-derived serine proteases. J Immunol 183:7592–7601. doi:10.4049/jimmunol.0803902. 19917683

[58] Unlu A, Tanriseven A, Sezen IY, Celik A 2015 A new lipase as a pharmaceutical target for battling infections caused by *Staphylococcus aureus*: gene cloning and biochemical characterization. Biotechnol Appl Biochem 62:642–651. doi:10.1002/bab.1316. 25385356

[59] Park H, Bae SH, Park Y, Choi HS, Suh HJ 2015 Lipase-mediated lipid removal from propolis extract and its antiradical and antimicrobial activity. J Sci Food Agric 95:1697–1705. doi:10.1002/jsfa.6874. 25123816

[60] Cadieux B, Vijayakumaran V, Bernards MA, McGavin MJ, Heinrichs DE 2014 Role of lipase from community-associated methicillin-resistant *Staphylococcus aureus* strain USA300 in hydrolyzing triglycerides into growth-inhibitory free fatty acids. J Bacteriol 196:4044–4056. doi:10.1128/JB.02044-14. 25225262 PMC4248881

[61] Saising J, Singdam S, Ongsakul M, Voravuthikunchai SP 2012 Lipase, protease, and biofilm as the major virulence factors in staphylococci isolated from acne lesions. Biosci Trends 6:160–164. doi:10.5582/bst.2012.v6.4.160. 23006962

[62] Sibbald MJ, Ziebandt AK, Engelmann S, Hecker M, de Jong A, Harmsen HJ, Raangs GC, Stokroos I, Arends JP, Dubois JY, van Dijl JM 2006 Mapping the pathways to staphylococcal pathogenesis by comparative secretomics. Microbiol Mol Biol Rev 70:755–788. doi:10.1128/MMBR.00008-06. 16959968 PMC1594592

[63] Yu W, Kim HK, Rauch S, Schneewind O, Missiakas D 2017 Pathogenic conversion of coagulase-negative staphylococci. Microbes Infect 19:101–109. doi:10.1016/j.micinf.2016.12.002. 28012900 PMC5274588

[64] Dos Santos DC, Lange CC, Avellar-Costa P, Dos Santos KR, Brito MA, Giambiagi-deMarval M 2016 *Staphylococcus chromogenes*, a coagulase-negative *Staphylococcus* species that can clot plasma. J Clin Microbiol 54:1372–1375. doi:10.1128/JCM.03139-15. 26912749 PMC4844742

[65] Taponen S, Supré K, Piessens V, Van Coillie E, De Vliegher S, Koort JM 2012 *Staphylococcus agnetis* sp. nov., a coagulase-variable species from bovine subclinical and mild clinical mastitis. Int J Syst Evol Microbiol 62:61–65. doi:10.1099/ijs.0.028365-0. 21335502

[66] Kuipers A, Stapels DA, Weerwind LT, Ko YP, Ruyken M, Lee JC, van Kessel KP, Rooijakkers SH 2016 The *Staphylococcus aureus* polysaccharide capsule and Efb-dependent fibrinogen shield act in concert to protect against phagocytosis. Microbiology 162:1185–1194. doi:10.1099/mic.0.000293. 27112346 PMC4977062

[67] Marques VF, de Souza MMS, de Mendonca ECL, de Alencar TA, Pribul BR, Coelho SDD, Lasagno M, Reinoso EB 2013 Phenotypic and genotypic analysis of virulence in *Staphylococcus* spp. and its clonal dispersion as a contribution to the study of bovine mastitis. Pesq Vet Bras 33:161–170. (In Portuguese with English summary.) doi:10.1590/S0100-736X2013000200005.

[68] Misawa Y, Kelley KA, Wang X, Wang L, Park WB, Birtel J, Saslowsky D, Lee JC 2015 *Staphylococcus aureus* colonization of the mouse gastrointestinal tract is modulated by wall teichoic acid, capsule, and surface proteins. PLoS Pathog 11:e1005061. doi:10.1371/journal.ppat.1005061. 26201029 PMC4511793

[69] Camussone C, Rejf P, Pujato N, Schwab A, Marcipar I, Calvinho LF 2012 Genotypic and phenotypic detection of capsular polysaccharides in *Staphylococcus aureus* isolated from bovine intramammary infections in Argentina. Braz J Microbiol 43:1010–1014. doi:10.1590/S1517-838220120003000023. 24031920 PMC3768870

[70] Thakker M, Park JS, Carey V, Lee JC 1998 Staphylococcus aureus serotype 5 capsular polysaccharide is antiphagocytic and enhances bacterial virulence in a murine bacteremia model. Infect Immun 66:5183–5189. 9784520 10.1128/iai.66.11.5183-5189.1998 PMC108646

[71] McLoughlin RM, Solinga RM, Rich J, Zaleski KJ, Cocchiaro JL, Risley A, Tzianabos AO, Lee JC 2006 CD4^+^ T cells and CXC chemokines modulate the pathogenesis of *Staphylococcus aureus* wound infections. Proc Natl Acad Sci U S A 103:10408–10413. doi:10.1073/pnas.0508961103. 16801559 PMC1502471

[72] Tuchscherr L, Loffler B, Buzzola FR, Sordelli DO 2010 *Staphylococcus aureus* adaptation to the host and persistence: role of loss of capsular polysaccharide expression. Future Microbiol 5:1823–1832. doi:10.2217/fmb.10.147. 21155664

[73] Tuchscherr LP, Buzzola FR, Alvarez LP, Caccuri RL, Lee JC, Sordelli DO 2005 Capsule-negative *Staphylococcus aureus* induces chronic experimental mastitis in mice. Infect Immun 73:7932–7937. doi:10.1128/IAI.73.12.7932-7937.2005. 16299284 PMC1307038

[74] Baddour LM, Tayidi MM, Walker E, McDevitt D, Foster TJ 1994 Virulence of coagulase-deficient mutants of *Staphylococcus aureus* in experimental endocarditis. J Med Microbiol 41:259–263. doi:10.1099/00222615-41-4-259. 7932618

[75] Herbert S, Worlitzsch D, Dassy B, Boutonnier A, Fournier JM, Bellon G, Dalhoff A, Doring G 1997 Regulation of Staphylococcus aureus capsular polysaccharide type 5: CO_2_ inhibition in vitro and in vivo. J Infect Dis 176:431–438. doi:10.1086/514061. 9237709

[76] Lattar SM, Tuchscherr LP, Caccuri RL, Centron D, Becker K, Alonso CA, Barberis C, Miranda G, Buzzola FR, von Eiff C, Sordelli DO 2009 Capsule expression and genotypic differences among *Staphylococcus aureus* isolates from patients with chronic or acute osteomyelitis. Infect Immun 77:1968–1975. doi:10.1128/IAI.01214-08. 19273557 PMC2681770

[77] Piepers S, Peeters K, Opsomer G, Barkema HW, Frankena K, De Vliegher S 2011 Pathogen group specific risk factors at herd, heifer and quarter levels for intramammary infections in early lactating dairy heifers. Prev Vet Med 99:91–101. doi:10.1016/j.prevetmed.2011.02.010. 21411160

[78] Rooijakkers SHM, Ruyken M, Van Roon J, Van Kessel KPM, Van Strijp JAG, Van Wamel WJB 2006 Early expression of SCIN and CHIPS drives instant immune evasion by *Staphylococcus aureus*. Cell Microbiol 8:1282–1293. doi:10.1111/j.1462-5822.2006.00709.x. 16882032

[79] van Wamel WJB, Rooijakkers SHM, Ruyken M, van Kessel KPM, van Strijp JAG 2006 The innate immune modulators staphylococcal complement inhibitor and chemotaxis inhibitory protein of *Staphylococcus aureus* are located on β-hemolysin-converting bacteriophages. J Bacteriol 188:1310–1315. doi:10.1128/JB.188.4.1310-1315.2006. 16452413 PMC1367213

[80] Verkaik NJ, Benard M, Boelens HA, de Vogel CP, Nouwen JL, Verbrugh HA, Melles DC, van Belkum A, van Wamel WJ 2011 Immune evasion cluster-positive bacteriophages are highly prevalent among human *Staphylococcus aureus* strains, but they are not essential in the first stages of nasal colonization. Clin Microbiol Infect 17:343–348. doi:10.1111/j.1469-0691.2010.03227.x. 20370801

[81] McCarthy AJ, Lindsay JA 2013 *Staphylococcus aureus* innate immune evasion is lineage-specific: a bioinformatics study. Infect Genet Evol 19:7–14. doi:10.1016/j.meegid.2013.06.012. 23792184

[82] Sheldon JR, Heinrichs DE 2015 Recent developments in understanding the iron acquisition strategies of gram positive pathogens. FEMS Microbiol Rev 39:592–630. doi:10.1093/femsre/fuv009. 25862688

[83] Haley KP, Skaar EP 2012 A battle for iron: host sequestration and *Staphylococcus aureus* acquisition. Microbes Infect 14:217–227. doi:10.1016/j.micinf.2011.11.001. 22123296 PMC3785375

[84] Hammer ND, Skaar EP 2011 Molecular mechanisms of *Staphylococcus aureus* iron acquisition. Annu Rev Microbiol 65:129–147. doi:10.1146/annurev-micro-090110-102851. 21639791 PMC3807827

[85] Maresso AW, Schneewind O 2006 Iron acquisition and transport in *Staphylococcus aureus*. Biometals 19:193–203. doi:10.1007/s10534-005-4863-7. 16718604

[86] Beasley FC, Heinrichs DE 2010 Siderophore-mediated iron acquisition in the staphylococci. J Inorg Biochem 104:282–288. doi:10.1016/j.jinorgbio.2009.09.011. 19850350

[87] Tiedemann MT, Muryoi N, Heinrichs DE, Stillman MJ 2008 Iron acquisition by the haem-binding Isd proteins in *Staphylococcus aureus*: studies of the mechanism using magnetic circular dichroism. Biochem Soc Trans 36:1138–1143. doi:10.1042/BST0361138. 19021512

[88] Reniere ML, Skaar EP 2008 *Staphylococcus aureus* heme oxygenases are differentially regulated by iron and heme. Mol Microbiol 69:1304–1315. doi:10.1111/j.1365-2958.2008.06363.x. 18643935 PMC2597461

[89] Skaar EP, Schneewind O 2004 Iron-regulated surface determinants (Isd) of *Staphylococcus aureus*: stealing iron from heme. Microbes Infect 6:390–397. doi:10.1016/j.micinf.2003.12.008. 15101396

[90] Lindsay J, Riley T, Mee B 1994 Production of siderophore by coagulase-negative staphylococci and its relation to virulence. Eur J Clin Microbiol Infect Dis 13:1063–1066. doi:10.1007/BF02111829. 7889970

[91] Moraveji Z, Tabatabaei M, Aski HS, Khoshbakht R 2014 Characterization of hemolysins of *Staphylococcus* strains isolated from human and bovine, southern Iran. Iran J Vet Res 15:326–330. 27175125 PMC4789207

[92] Unal N, Askar S, Macun HC, Sakarya F, Altun B, Yildirim M 2012 Panton-Valentine leukocidin and some exotoxins of *Staphylococcus aureus* and antimicrobial susceptibility profiles of staphylococci isolated from milks of small ruminants. Trop Anim Health Prod 44:573–579. doi:10.1007/s11250-011-9937-7. 21800213

[93] Tseng CW, Biancotti JC, Berg BL, Gate D, Kolar SL, Muller S, Rodriguez MD, Rezai-Zadeh K, Fan X, Beenhouwer DO, Town T, Liu GY 2015 Increased susceptibility of humanized NSG mice to Panton-Valentine leukocidin and *Staphylococcus aureus* skin infection. PLoS Pathog 11:e1005292. doi:10.1371/journal.ppat.1005292. 26618545 PMC4664407

[94] Chen Y, Yeh AJ, Cheung GY, Villaruz AE, Tan VY, Joo HS, Chatterjee SS, Yu Y, Otto M 2015 Basis of virulence in a Panton-Valentine leukocidin-negative community-associated methicillin-resistant Staphylococcus aureus strain. J Infect Dis 211:472–480. doi:10.1093/infdis/jiu462. 25139021 PMC4296174

[95] Fijalkowski K, Struk M, Karakulska J, Paszkowska A, Giedrys-Kalemba S, Masiuk H, Czernomysy-Furowicz D, Nawrotek P 2014 Comparative analysis of superantigen genes in *Staphylococcus xylosus* and *Staphylococcus aureus* isolates collected from a single mammary quarter of cows with mastitis. J Microbiol 52:366–372. doi:10.1007/s12275-014-3436-2. 24723103

[96] Ates LS, Houben EN, Bitter W 2016 Type VII secretion: a highly versatile secretion system. Microbiol Spectr 4:VMBF-0011-2015. doi:10.1128/microbiolspec.VMBF-0011-2015. 26999398

[97] Warne B, Harkins CP, Harris SR, Vatsiou A, Stanley-Wall N, Parkhill J, Peacock SJ, Palmer T, Holden MT 2016 The Ess/Type VII secretion system of *Staphylococcus aureus* shows unexpected genetic diversity. BMC Genomics 17:222. doi:10.1186/s12864-016-2426-7. 26969225 PMC4788903

[98] Houben EN, Bestebroer J, Ummels R, Wilson L, Piersma SR, Jimenez CR, Ottenhoff TH, Luirink J, Bitter W 2012 Composition of the type VII secretion system membrane complex. Mol Microbiol 86:472–484. doi:10.1111/j.1365-2958.2012.08206.x. 22925462

[99] Cao Z, Casabona MG, Kneuper H, Chalmers JD, Palmer T 2016 The type VII secretion system of *Staphylococcus aureus* secretes a nuclease toxin that targets competitor bacteria. Nat Microbiol 2:16183. doi:10.1038/nmicrobiol.2016.183. 27723728 PMC5325307

[100] Kneuper H, Cao ZP, Twomey KB, Zoltner M, Jager F, Cargill JS, Chalmers J, van der Kooi-Pol MM, van Dijl JM, Ryan RP, Hunter WN, Palmer T 2014 Heterogeneity in ess transcriptional organization and variable contribution of the Ess/Type VII protein secretion system to virulence across closely related *Staphylococcus aureus* strains. Mol Microbiol 93:928–943. doi:10.1111/mmi.12707. 25040609 PMC4285178

[101] Wang R, Braughton KR, Kretschmer D, Bach T-HL, Queck SY, Li M, Kennedy AD, Dorward DW, Klebanoff SJ, Peschel A, DeLeo FR, Otto M 2007 Identification of novel cytolytic peptides as key virulence determinants for community-associated MRSA. Nat Med 13:1510. doi:10.1038/nm1656. 17994102

[102] Otto M 2014 Phenol-soluble modulins. Int J Med Microbiol 304:164–169. doi:10.1016/j.ijmm.2013.11.019. 24447915 PMC4014003

[103] Wang R, Khan BA, Cheung GYC, Bach T-HL, Jameson-Lee M, Kong K-F, Queck SY, Otto M 2011 *Staphylococcus epidermidis* surfactant peptides promote biofilm maturation and dissemination of biofilm-associated infection in mice. J Clin Invest 121:238–248. doi:10.1172/JCI42520. 21135501 PMC3007140

[104] Otto M 2009 *Staphylococcus epidermidis* — the 'accidental' pathogen. Nat Rev Microbiol 7:555. doi:10.1038/nrmicro2182. 19609257 PMC2807625

[105] Towle KM, Lohans CT, Miskolzie M, Acedo JZ, van Belkum MJ, Vederas JC 2016 Solution structures of phenol-soluble modulins alpha1, alpha3, and beta2, virulence factors from *Staphylococcus aureus*. Biochemistry 55:4798–4806. doi:10.1021/acs.biochem.6b00615. 27525453

[106] Periasamy S, Chatterjee SS, Cheung GY, Otto M 2012 Phenol-soluble modulins in staphylococci: what are they originally for? Commun Integr Biol 5:275–277. doi:10.4161/cib.19420. 22896791 PMC3419113

[107] Cheung GY, Joo HS, Chatterjee SS, Otto M 2014 Phenol-soluble modulins – critical determinants of staphylococcal virulence. FEMS Microbiol Rev 38:698–719. doi:10.1111/1574-6976.12057. 24372362 PMC4072763

[108] Mello PL, Moraes Riboli DF, Pinheiro L, de Almeida Martins L, Vasconcelos Paiva Brito MA, Ribeiro de Souza da Cunha MDL 2016 Detection of enterotoxigenic potential and determination of clonal profile in Staphylococcus aureus and coagulase-negative staphylococci isolated from bovine subclinical mastitis in different Brazilian states. Toxins (Basel) 8:104. doi:10.3390/toxins8040104. 27092525 PMC4848630

[109] Park JY, Fox LK, Seo KS, McGuire MA, Park YH, Rurangirwa FR, Sischo WM, Bohach GA 2011 Detection of classical and newly described staphylococcal superantigen genes in coagulase-negative staphylococci isolated from bovine intramammary infections. Vet Microbiol 147:149–154. doi:10.1016/j.vetmic.2010.06.021. 20667668 PMC3689430

[110] da Silva SDSP, Cidral TA, Soares MJDSS, de Melo MCN 2015 Enterotoxin-encoding genes in Staphylococcus spp. from food handlers in a university restaurant. Foodborne Pathog Dis 12:921–925. doi:10.1089/fpd.2015.1941. 26352253

[111] Bergevin M, Marion A, Farber D, Golding GR, Levesque S 2017 Severe MRSA enterocolitis caused by a strain harboring enterotoxins D, G, and I. Emerg Infect Dis 23:865–867. doi:10.3201/eid2305.161644. 28418301 PMC5403038

[112] Bertelloni F, Fratini F, Ebani VV, Galiero A, Turchi B, Cerri D 2015 Detection of genes encoding for enterotoxins, TSST-1, and biofilm production in coagulase-negative staphylococci from bovine bulk tank milk. Dairy Sci Technol 95:341–352. doi:10.1007/s13594-015-0214-9.

[113] Piechota M, Kot B, Zdunek E, Mitrus J, Wicha J, Wolska MK, Sachanowicz K 2014 Distribution of classical enterotoxin genes in staphylococci from milk of cows with- and without mastitis and the cowshed environment. Pol J Vet Sci 17:407–411. doi:10.2478/pjvs-2014-0058. 25286646

[114] Bulajic S, Colovic S, Misic D, Djordjevic J, Savic-Radovanovic R, Asanin J, Ledina T 2017 Enterotoxin production and antimicrobial susceptibility in staphylococci isolated from traditional raw milk cheeses in Serbia. J Environ Sci Health B 52:864–870. doi:10.1080/03601234.2017.1361764. 28949803

[115] Nia Y, Mutel I, Assere A, Lombard B, Auvray F, Hennekinne JA 2016 Review over a 3-year period of European Union proficiency tests for detection of staphylococcal enterotoxins in food matrices. Toxins (Basel) 8:107. doi:10.3390/toxins8040107. 27089364 PMC4848633

[116] Bastos CP, Bassani MT, Mata MM, Lopes GV, da Silva WP 2017 Prevalence and expression of staphylococcal enterotoxin genes in *Staphylococcus aureus* isolated from food poisoning outbreaks. Can J Microbiol 63:834–840. doi:10.1139/cjm-2017-0316. 28820948

[117] Schubert J, Podkowik M, Bystroń J, Bania J 2017 Production of staphylococcal enterotoxins D and R in milk and meat juice by *Staphylococcus aureus* strains. Foodborne Pathog Dis 14:223–230. doi:10.1089/fpd.2016.2210. 28072918

[118] Andjelkovic M, Tsilia V, Rajkovic A, De Cremer K, Van Loco J 2016 Application of LC-MS/MS MRM to determine staphylococcal enterotoxins (SEB and SEA) in milk. Toxins (Basel) 8:118. doi:10.3390/toxins8040118. 27104569 PMC4848643

[119] Nemati M, Hermans K, Vancraeynest D, De Vliegher S, Sampimon OC, Baele M, De Graef EM, Pasmans F, Haesebrouck F 2008 Screening of bovine coagulase-negative staphylococci from milk for superantigen-encoding genes. Vet Rec 163:740–743. 19103615

[120] Chaves-Moreno D, Wos-Oxley ML, Jáuregui R, Medina E, Oxley APA, Pieper DH 2016 Exploring the transcriptome of Staphylococcus aureus in its natural niche. Sci Rep 6:33174. doi:10.1038/srep33174. 27641137 PMC5027550

[121] Taur Y, Pamer EG 2013 The intestinal microbiota and susceptibility to infection in immunocompromised patients. Curr Opin Infect Dis 26:332–337. doi:10.1097/QCO.0b013e3283630dd3. 23806896 PMC4485384

[122] Yoong P, Torres VJ 2015 Counter inhibition between leukotoxins attenuates *Staphylococcus aureus* virulence. Nat Commun 6:8125. doi:10.1038/ncomms9125. 26330208 PMC4562310

[123] Reyher KK, Dufour S, Barkema HW, Des Coteaux L, Devries TJ, Dohoo IR, Keefe GP, Roy JP, Scholl DT 2011 The National Cohort of Dairy Farms–a data collection platform for mastitis research in Canada. J Dairy Sci 94:1616–1626. doi:10.3168/jds.2010-3180. 21338829

[124] Nobrega DB, Naushad S, Naqvi SA, Condas LAZ, Saini V, Kastelic JP, Luby C, De Buck J, Barkema HW 2018 Prevalence and genetic basis of antimicrobial resistance in non-aureus staphylococci isolated from Canadian dairy herds. Front Microbiol 9:256. doi:10.3389/fmicb.2018.00256. 29503642 PMC5820348

[125] Carson DA, Barkema HW, Naushad S, De Buck J 2017 Bacteriocins of non-aureus staphylococci isolated from bovine milk. Appl Environ Microbiol 83:e01015-17. doi:10.1128/AEM.01015-17. 28667105 PMC5561277

[126] Koster J, Rahmann S 2012 Snakemake—a scalable bioinformatics workflow engine. Bioinformatics 28:2520–2522. doi:10.1093/bioinformatics/bts480. 22908215

[127] Martin M 2011 Cutadapt removes adapter sequences from high-throughput sequencing reads. EMBnetjournal 17:10–12.

[128] Nurk S, Bankevich A, Antipov D, Gurevich AA, Korobeynikov A, Lapidus A, Prjibelski AD, Pyshkin A, Sirotkin A, Sirotkin Y, Stepanauskas R, Clingenpeel SR, Woyke T, McLean JS, Lasken R, Tesler G, Alekseyev MA, Pevzner PA 2013 Assembling single-cell genomes and mini-metagenomes from chimeric MDA products. J Comput Biol 20:714–737. doi:10.1089/cmb.2013.0084. 24093227 PMC3791033

[129] Bankevich A, Nurk S, Antipov D, Gurevich AA, Dvorkin M, Kulikov AS, Lesin VM, Nikolenko SI, Pham S, Prjibelski AD, Pyshkin AV, Sirotkin AV, Vyahhi N, Tesler G, Alekseyev MA, Pevzner PA 2012 SPAdes: a new genome assembly algorithm and its applications to single-cell sequencing. J Comput Biol 19:455–477. doi:10.1089/cmb.2012.0021. 22506599 PMC3342519

[130] Gurevich A, Saveliev V, Vyahhi N, Tesler G 2013 QUAST: quality assessment tool for genome assemblies. Bioinformatics 29:1072–1075. doi:10.1093/bioinformatics/btt086. 23422339 PMC3624806

[131] Seemann T 2014 Prokka: rapid prokaryotic genome annotation. Bioinformatics 30:2068–2069. doi:10.1093/bioinformatics/btu153. 24642063

[132] Chen L, Zheng D, Liu B, Yang J, Jin Q 2016 VFDB 2016: hierarchical and refined dataset for big data analysis–10 years on. Nucleic Acids Res 44:D694–D697. doi:10.1093/nar/gkv1239. 26578559 PMC4702877

[133] Wattam AR, Abraham D, Dalay O, Disz TL, Driscoll T, Gabbard JL, Gillespie JJ, Gough R, Hix D, Kenyon R, Machi D, Mao C, Nordberg EK, Olson R, Overbeek R, Pusch GD, Shukla M, Schulman J, Stevens RL, Sullivan DE, Vonstein V, Warren A, Will R, Wilson MJ, Yoo HS, Zhang C, Zhang Y, Sobral BW 2014 PATRIC, the bacterial bioinformatics database and analysis resource. Nucleic Acids Res 42:D581–D591. doi:10.1093/nar/gkt1099. 24225323 PMC3965095

[134] The UniProt Consortium. 2017 UniProt: the universal protein knowledgebase. Nucleic Acids Res 45:D158–D169. doi:10.1093/nar/gkw1099. 27899622 PMC5210571

[135] Camacho C, Coulouris G, Avagyan V, Ma N, Papadopoulos J, Bealer K, Madden TL 2009 BLAST+: architecture and applications. BMC Bioinformatics 10:421. doi:10.1186/1471-2105-10-421. 20003500 PMC2803857

[136] Li J, Tai C, Deng Z, Zhong W, He Y, Ou HY 2017 VRprofile: gene-cluster-detection-based profiling of virulence and antibiotic resistance traits encoded within genome sequences of pathogenic bacteria. Brief Bioinform 19:566–574. doi:10.1093/bib/bbw141. 28077405

[137] Pearson WR 2013 Selecting the right similarity-scoring matrix. Curr Protoc Bioinformatics 43:3.5.1–3.5.9. doi:10.1002/0471250953.bi0305s43. 24509512 PMC3848038

[138] Rost B 1999 Twilight zone of protein sequence alignments. Protein Eng 12:85–94. doi:10.1093/protein/12.2.85. 10195279

[139] Pearson WR 2013 An introduction to sequence similarity (“homology”) searching. Curr Protoc Bioinformatics Chapter 3:Unit 3.1. doi:10.1002/0471250953.bi0301s27. PMC3820096 23749753

[140] R Development Core Team. 2016 R: a language and environment for statistical computing. R Foundation for Statistical Computing, Vienna, Austria.

[141] Hadley W, Romain F, Lionel H, Müller K 2017 dplyr: a grammar of data manipulation. R package version 0.7.2.

[142] Ward N, Moreno-Hagelsieb G 2014 Quickly finding orthologs as reciprocal best hits with BLAT, LAST, and UBLAST: how much do we miss? PLoS One 9:e101850. doi:10.1371/journal.pone.0101850. 25013894 PMC4094424

[143] Meyer D, Zeileis D, Hornik K 2017 *vcd*: visualizing categorical data. R package version 1.4-4.

[144] Wei T, Simko V 2017 R package “corrplot”: visualization of a correlation matrix (version 0.84). Available from https://github.com/taiyun/corrplot.

[145] Christensen RHB 2015 Ordinal - regression models for ordinal data. R package version 2015.6-28. Available from https://cran.r-project.org/web/packages/ordinal/index.html.

[146] Pedregosa F, Varoquaux G, Varoquaux G, Michel V, Thirion B, Grisel O, Blondel M, Prettenhofer P, Weiss R, Dubourg V, Vanderplas J, Passos A, Cournapeau D, Brucher M, Perrot M, Duchesnay É 2011 Scikit-learn: machine learning in Python. J Machine Learning Res 12:2825–2830.

[147] van der Maaten L, Hinton G 2008 Visualizing data using t-SNE. J Machine Learning Res 9:2579–2605.

