# Supplementary figures and images for: Comprehensive Virulence Gene Profiling of Bovine Non-aureus Staphylococci Based on Whole-Genome Sequencing Data
Source: mSystems. 2019 Mar 5;4(2):e00098-18. doi: 10.1128/mSystems.00098-18 (PMC6401416; doi:10.1128/mSystems.00098-18)

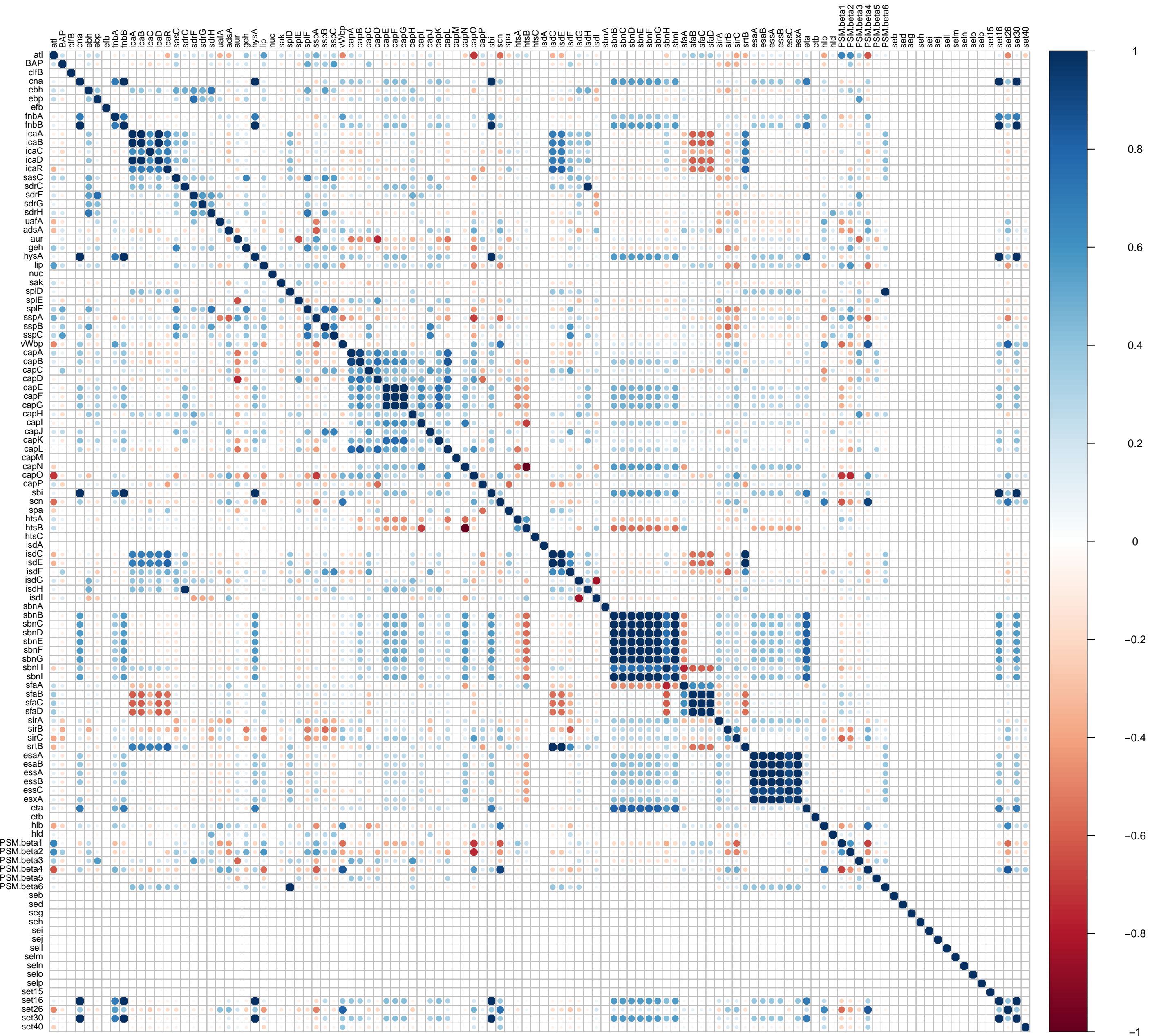

Supplement: FIG S1 [file mSystems.00098-18-sf001.pdf]

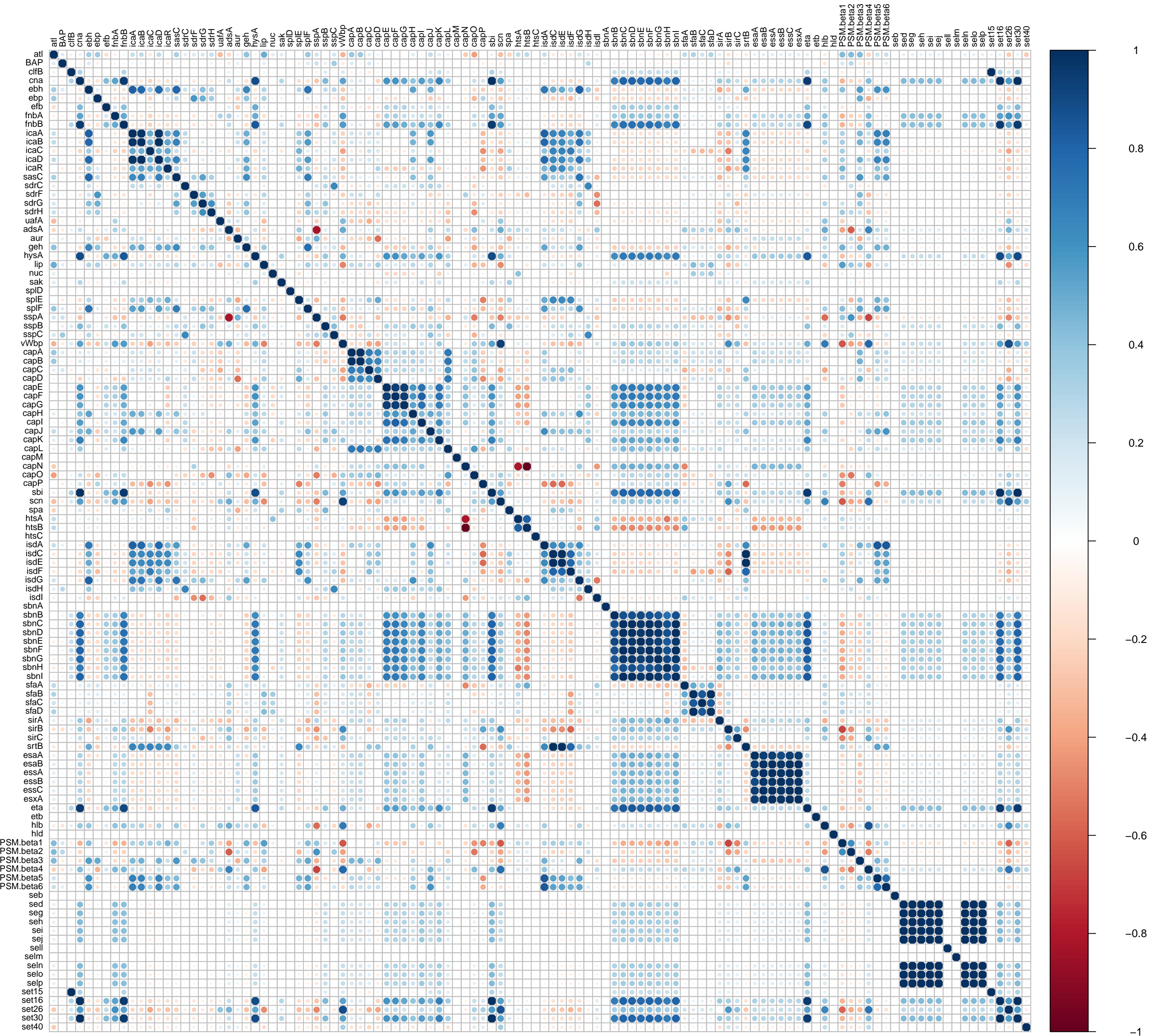

Supplement: FIG S2 [file mSystems.00098-18-sf002.pdf]

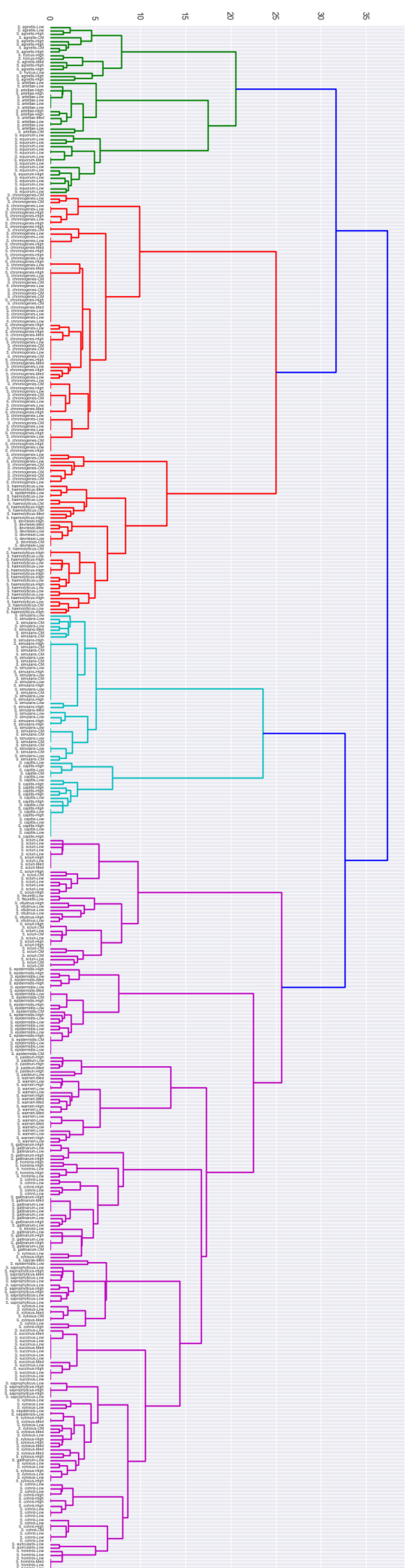

Supplement: FIG S3 [file mSystems.00098-18-sf003.pdf]

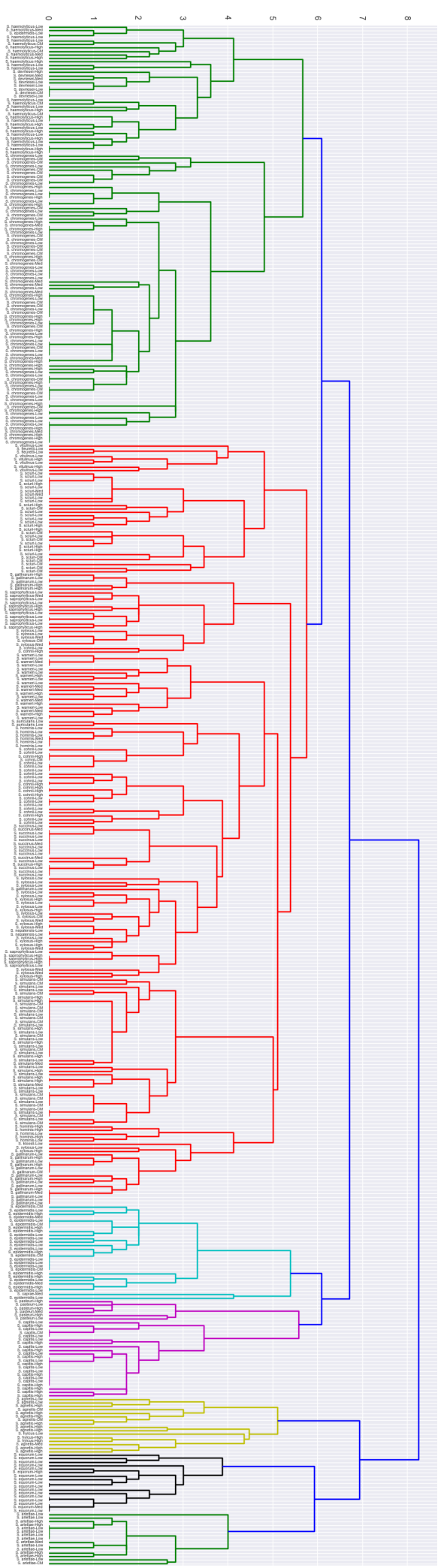

Supplement: FIG S4 [file mSystems.00098-18-sf004.pdf]
